# Supplementary material for: Synthesis and macrocyclization-induced emission enhancement of benzothiadiazole-based macrocycle
Source: Nat Commun. 2022 May 23;13:2850. doi: 10.1038/s41467-022-30121-0 (PMC9126912; doi:10.1038/s41467-022-30121-0)
Supplement: Supplementary file 1 — Supplementary Information [file 41467_2022_30121_MOESM1_ESM.pdf]

Supplementary Information

Synthesis and Macrocyclization-Induced Emission Enhancement of Benzothiadiazole-based Macrocycle

Shuo Li,[1] Kun Liu,[2] Xue-Chen Feng,[2] Zhao-Xian Li,[2] Zhi-Yuan Zhang,[2] Bin Wang,[2] Minjie Li,[1] Yue-Ling Bai,*[1] Lei Cui,*[1] and Chunju Li*[1,2]

[1] College of Sciences, Center for Supramolecular Chemistry and Catalysis, Shanghai University, Shanghai 200444 (P. R. China). yuelingbai@shu.edu.cn; cuilei@shu.edu.cn
[2] Tianjin Key Laboratory of Structure and Performance for Functional Molecules, College of Chemistry, Tianjin Normal University, Tianjin 300387 (P. R. China). cjli@shu.edu.cn

Table of Contents

1. Supplementary methods S3

1.1 Experimental details S3

1.2 ^{1}H NMR, ^{13}C NMR, high resolution mass spectra (HRMS) S9

2. Supplementary Discussion S24

2.1 Photophysical properties S24

2.2 Crystallography data S31

2.3 Theoretical calculation S35

2.4 Thermogravimetric analysis S41

2.5 Electroluminescence S41

3. Supplementary References S44

1. Supplementary methods

1.1 Experimental details

BT-M. Under the protection of N_2 atmosphere, 4,7-dibromo-2,1,3-benzothiadiazole (2.94 g, 10.0 mmol), 2,4-dimethoxybenzeneboronic acid (5.46 g, 30.0 mmol), and tetrakis(triphenylphosphine)palladium(0) (1.16 g, 1.00 mmol) were dissolved in tetrahydrofuran (150 mL). The sodium carbonate (3.18 g, 30.0 mmol) in water (15 mL) was added into the solution and stirred for 24 h at 85 ℃. Upon cooling to room temperature, water (100 mL), dichloromethane (100 mL) was added and stirred. After filtration of the solution, the solution was partitioned between dichloromethane and water. The product was extracted from the organic layer. The aqueous layer was further extracted twice with dichloromethane (100 mL). The combined organic layer was dried over anhydrous Na_2SO_4 and evaporated under reduced pressure. The product was purified by column chromatography on silica gel (eluent: 3/1, v/v, dichloromethane : petroleum ether) to give yellow product 4,7-bis(2,4-dimethoxyphenyl)-2,1,3-benzothiadiazole (3.34 g, 82%) as a yellow solid. m.p. 181-182 ^{o}C; ^{1}H NMR (500 MHz, $CDCl_3$, 298 K) δ 7.72 (s, 2H), 7.56 (d, *J* = 10.0 Hz, 2H), 6.72-6.70 (m, 4H), 3.93 (s, 6H), 3.83 (s, 6H). ^{13}C NMR (125 MHz, $CDCl_3$, 298 K) δ 161.1, 158.2, 154.8, 132.4, 130.1, 129.8, 119.5, 104.7, 99.3, 55.7, 55.5. HRMS (ESI) m/z: $[M+H]^+$ calcd for $[C_{22}H_{21}N_2O_4S]^+$, 409.1217; found, 409.1218.

BT-LC. To the solution of 4,7-bis(2,4-dimethoxyphenyl)-2,1,3-benzothiadiazole (4.00 g, 10.0 mmol) in dichloromethane (300 mL) was added paraformaldehyde (0.900 g, 30.0 mmol). Boron trifluoride diethyl etherate (1.30 ml, 10.0 mmol) was then added to the reaction mixture. The mixture was stirred at 25 ^{o}C for 25 minutes. Then the reaction was quenched by addition of 200 mL saturated aqueous $NaHCO_3$. The solution was partitioned between dichloromethane and saturated aqueous $NaHCO_3$. The product was extracted from the organic layer. The aqueous layer was further extracted twice with dichloromethane (100 mL). The combined organic layer was dried over anhydrous Na_2SO_4 and concentrated. The product was purified by column chromatography on silica gel (eluent : dichloromethane) to obtain product BT-LC (2.18 g, 52%) as a yellow solid. m.p. 225-226 ^{o}C; ^{1}H NMR (500 MHz, CD_2Cl_2, 298 K) δ 7.68 (s , 6H), 7.33 (s, 6H), 6.74 (s, 6H), 4.11 (s, 6H), 4.01 (s, 18H), 3.85 (s, 18H). ^{13}C NMR (125 MHz, CD_2Cl_2, 298 K) δ 158.5, 156.9, 154.7, 133.2, 130.3, 129.4, 120.7, 118.1, 95.6, 56.5, 55.6, 27.7. HRMS (ESI) m/z: $[M+H]^+$ calcd for $[C_{69}H_{61}N_6O_{12}S_3]^+$, 1261.3504; found, 1261.3513.

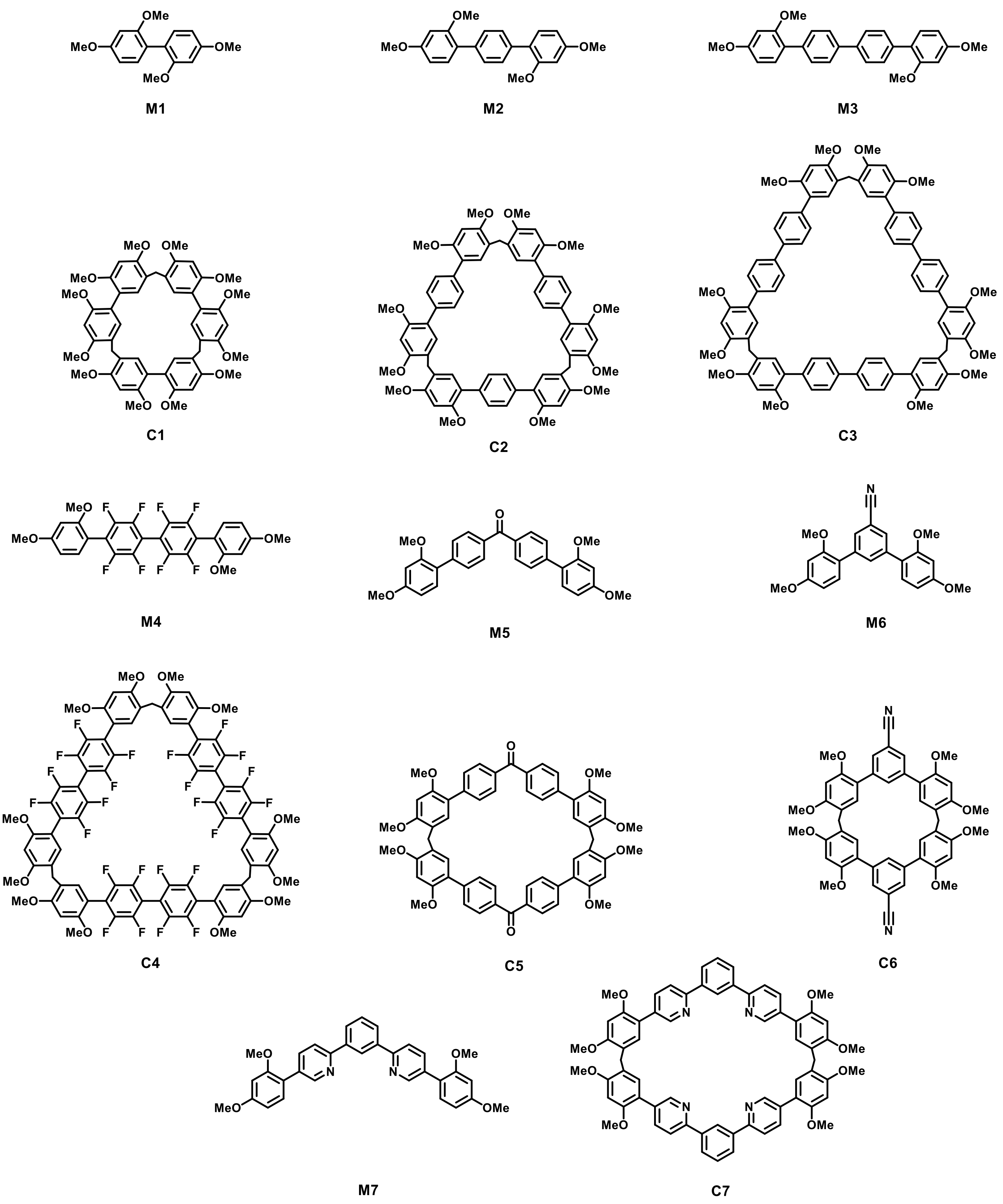

Supplementary Figure 1. Chemical structure of other monomers and corresponding macrocycles.

M1-M3 and C1-C3. The compounds were obtained according to our previous work.[1,2]

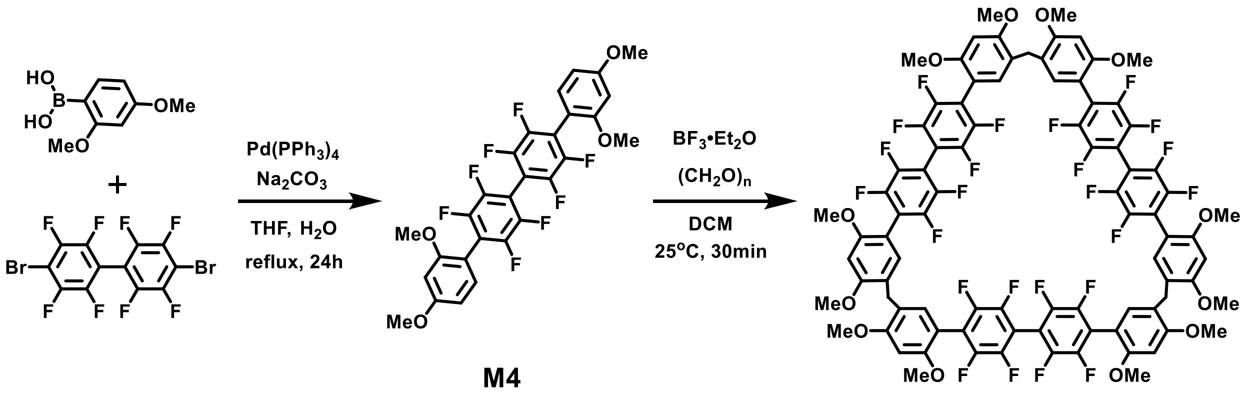

M4. Under the protection of N_2 atmosphere, 4,4'-dibromooctafluorobiphenyl (4.56 g, 10.0 mmol), 2,4-dimethoxybenzeneboronic acid (4.55 g, 25.0 mmol), and tetrakis(triphenylphosphine)palladium(0) (1.16 g, 1.00 mmol) were dissolved in tetrahydrofuran (160 mL). The sodium carbonate (4.24 g, 40.0 mmol) in water (25 mL) was added into the solution and stirred for 24 h at 85 ℃. Upon cooling to room temperature, water (120 mL), dichloromethane (120 mL) was added and stirred. After filtration of the solution, the solution was partitioned between dichloromethane and water. The product was extracted from the organic layer. The aqueous layer was further extracted twice with dichloromethane (120 mL). The combined organic layer was dried over anhydrous Na_2SO_4 and evaporated under reduced pressure. The product was purified by column chromatography on silica gel (eluent: 4/1, v/v, dichloromethane : petroleum ether) to give product M4 (4.33g, 76%) as a white solid. m.p. 198-199 °C; ^{1}H NMR (400 MHz, $CDCl_3$, 298 K) δ 7.25 (d, J = 8.0 Hz, 2H), 6.66-6.63 (m, 4H), 3.89 (s, 6H), 3.85 (s, 6H). ^{13}C NMR (100 MHz, $CDCl_3$, 298 K) δ 162.5, 158.5, 145.5, 143.5, 132.4, 119.7, 108.5, 106.1, 105.1, 99.1, 55.9, 55.6. HRMS (ESI) m/z: $[M+H]^+$ calcd for $[C_{28}H_{19}F_8O_4]^+$, 571.1150; found, 571.1156.

C4. To the solution of M4 (2.85g, 5.00 mmol) in dichloromethane (200 mL) was added paraformaldehyde (0.450 g, 15.0 mmol). Boron trifluoride diethyl etherate (0.650 ml, 5.00 mmol) was then added to the reaction mixture. The mixture was stirred at 25 °C for 30 minutes. Then the reaction was quenched by addition of 200 mL saturated aqueous $NaHCO_3$. The solution was partitioned between dichloromethane and saturated aqueous $NaHCO_3$. The product was extracted from the organic layer. The aqueous layer was further extracted twice with dichloromethane (120 mL). The combined organic layer was dried over anhydrous Na_2SO_4 and concentrated. The product was purified by column chromatography on silica gel (eluent : dichloromethane: ethyl acetate = 3:1) to obtain product C4 (1.05 g, 36%) as a white solid. m.p. >320°C; ^{1}H NMR (400 MHz, DMSO, 353 K) δ 8.39-6.68 (m , 12H), 3.92 (s, 18H), 3.86 (s, 18H), 3.81 (s, 6H). ^{13}C NMR (100 MHz, $CDCl_3$, 298 K) δ 159.8, 156.9, 145.6, 143.1, 132.5, 131.0, 128.9, 121.4, 119.7, 107.4, 105.9, 95.7, 56.0, 55.8, 28.1. HRMS (ESI) m/z: $[M+H]^+$ calcd for $[C_{87}H_{55}F_{24}O_{12}]^+$, 1747.3305; found, 1747.3251.

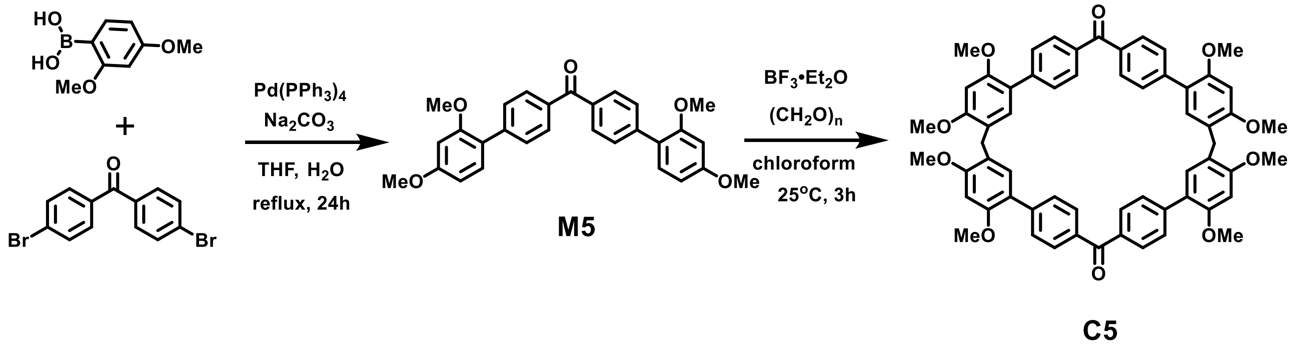

M5. Under the protection of N_2 atmosphere, 4,4'-dibromobenzophenone (6.80 g, 20.0 mmol), 2,4-dimethoxybenzeneboronic acid (9.10 g, 50.0 mmol), and tetrakis(triphenylphosphine)palladium(0) (2.32 g, 2.00 mmol) were dissolved in tetrahydrofuran (350 mL). The sodium carbonate (8.48 g, 80.0 mmol) in water (50 mL) was added into the solution and stirred for 24 h at 85 ℃. Upon cooling to room temperature, water (200 mL), dichloromethane (200 mL) was added and stirred. After filtration of the solution, the solution was partitioned between dichloromethane and water. The product was extracted from the organic layer. The aqueous layer was further extracted twice with dichloromethane (200 mL). The combined organic layer was dried over anhydrous Na_2SO_4 and evaporated under reduced pressure. The product was purified by column chromatography on silica gel (eluent: 4/1, v/v, dichloromethane : petroleum ether) to give product M5 (8.54g, 94%) as a white solid. m.p. 175-176 °C; ^{1}H NMR (600 MHz, $CDCl_3$, 298 K) δ 7.90 (d, J = 6.00 Hz, 4H), 7.65 (d, J = 6.00 Hz, 4H), 7.32 (d, J = 6.00 Hz, 2H), 6.62-6.59 (m, 4H), 3.87 (s, 6H), 3.84 (s, 6H). ^{13}C NMR (150 MHz, $CDCl_3$, 298 K) δ 196.3, 161.0, 157.7, 142.8, 135.8, 131.5, 130.0, 129.3, 122.5, 105.0, 99.2, 55.7, 55.6. HRMS (ESI) m/z: $[M+H]^+$ calcd for $[C_{29}H_{27}O_5]^+$, 455.1853; found, 455.1860.

C5. To the solution of M5 (0.910 g, 2.00 mmol) in chloroform (100 mL) was added paraformaldehyde (0.180 g, 6.00 mmol). Boron trifluoride diethyl etherate (0.260 ml, 2.00 mmol) was then added to the reaction mixture. The mixture was stirred at 25 °C for 3 hours. Then the reaction was quenched by addition of 100 mL saturated aqueous $NaHCO_3$. The solution was partitioned between dichloromethane and saturated aqueous $NaHCO_3$. The product was extracted from the organic layer. The aqueous layer was further extracted twice with dichloromethane (100 mL). The combined organic layer was dried over anhydrous Na_2SO_4 and concentrated. The product was purified by column chromatography on silica gel (eluent : dichloromethane: ethyl acetate = 8:1) to obtain product C5 (0.056 g, 6%) as a white solid. m.p. >320°C; ^{1}H NMR (600 MHz, $CDCl_3$, 298 K) δ 7.81 (d, J = 12.0 Hz, 8H), 7.57 (d, J = 6.00 Hz, 8H), 7.03 (s, 4H), 6.57 (s, 4H), 3.94 (s, 4H), 3.90 (s, 12H), 3.84 (s, 12H). ^{13}C NMR (150 MHz, $CDCl_3$, 298 K) δ 196.5, 158.4, 156.1, 142.9, 135.6, 131.9, 130.1, 129.3, 121.7, 121.3, 96.0, 56.1, 55.9, 27.3. HRMS (ESI) m/z: $[M+H]^+$ calcd for $[C_{60}H_{53}O_{10}]^+$, 933.3633; found, 933.3648.

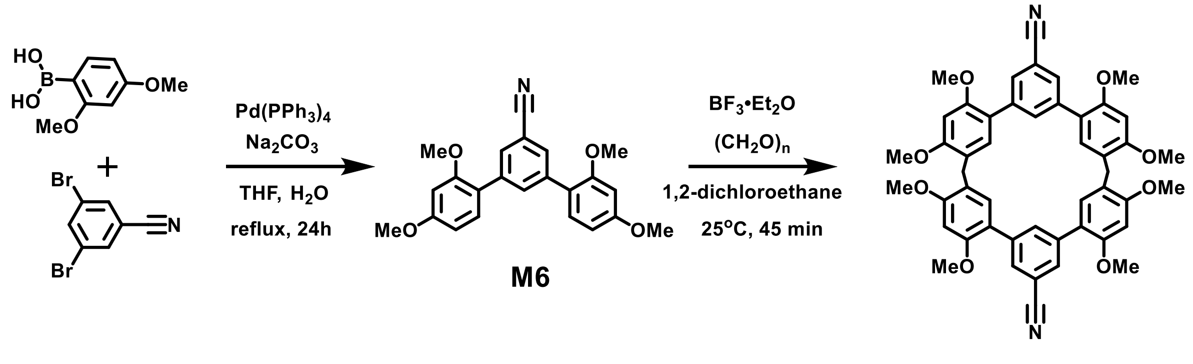

M6. Under the protection of N_2 atmosphere, 3,5-dibromobenzonitrile (5.22 g, 20.0 mmol), 2,4-dimethoxybenzeneboronic acid (9.10 g, 50.0 mmol), and tetrakis(triphenylphosphine)palladium(0) (2.32 g, 2.00 mmol) were dissolved in tetrahydrofuran (300 mL). The sodium carbonate (6.36 g, 60.0 mmol) in water (40 mL) was added into the solution and stirred for 24 h at 85 ℃. Upon cooling to room temperature, water (150 mL), dichloromethane (150 mL) was added and stirred. After filtration of the solution, the solution was partitioned between dichloromethane and water. The product was extracted from the organic layer. The aqueous layer was further extracted twice with dichloromethane (150 mL). The combined organic layer was dried over anhydrous Na_2SO_4 and evaporated under reduced pressure. The product was purified by column chromatography on silica gel (eluent: 4/1, v/v, dichloromethane : petroleum ether) to give product M5 (6.83 g, 91%) as a white solid. m.p. 185-186 °C; ^{1}H NMR (400 MHz, $CDCl_3$, 298 K) δ 7.82 (s, 1H), 7.74 (s, 2H), 7.26 (d, J = 8.0 Hz, 2H), 6.60-6.58 (m, 4H), 3.87 (s, 6H), 3.83 (s, 6H). ^{13}C NMR (100 MHz, $CDCl_3$, 298 K) δ 161.1, 157.5, 139.3, 134.9, 131.3, 131.1, 121.4, 119.7, 111.7, 105.0, 99.0, 55.7, 55.6. HRMS (ESI) m/z: $[M+H]^+$ calcd for $[C_{23}H_{22}NO_4]^+$, 376.1543; found, 376.1540.

C6. To the solution of M6 (1.86 g, 5.0 mmol) in 1,2-dichloroethane (150 mL) was added paraformaldehyde (0.450 g, 15.00 mmol). Boron trifluoride diethyl etherate (0.650 ml, 5.00 mmol) was then added to the reaction mixture. The mixture was stirred at 25 °C for 45 minutes. Then the reaction was quenched by addition of 150 mL saturated aqueous $NaHCO_3$. The solution was partitioned between dichloromethane and saturated aqueous $NaHCO_3$. The product was extracted from the organic layer. The aqueous layer was further extracted twice with dichloromethane (150 mL). The combined organic layer was dried over anhydrous Na_2SO_4 and concentrated. The product was purified by column chromatography on silica gel (eluent : dichloromethane: ethyl acetate = 20:1) to obtain product C6 (0.213 g, 11%) as a white solid. m.p. >320°C; ^{1}H NMR (400 MHz, $CDCl_3$, 298 K) δ 7.83 (s, 4H), 7.26 (s, 2H), 6.77 (s, 4H), 6.56 (s, 4H), 3.88 (s, 12H), 3.86 (s, 12H), 3.82 (s, 4H). ^{13}C NMR (100 MHz, $CDCl_3$, 298 K) δ 158.6, 155.7, 139.9, 134.0, 131.5, 131.4, 121.0, 119.9, 110.9, 95.3, 55.8, 55.7, 28.5. HRMS (ESI) m/z: $[M+Na]^+$ calcd for $[C_{48}H_{42}N_2O_8]^+$, 797.2833; found, 797.2839.

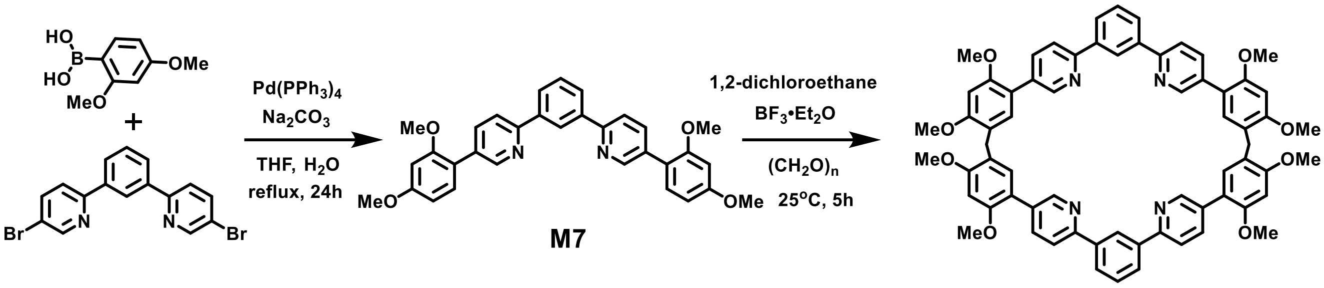

C7

M7. Under the protection of N_2 atmosphere, 1,3-bis(5-bromopyridin-2-yl)benzene (3.88 g, 10.0 mmol), 2,4-dimethoxybenzeneboronic acid (5.46 g, 30.0 mmol), and tetrakis(triphenylphosphine)palladium(0) (1.16 g, 1.00 mmol) were dissolved in tetrahydrofuran (200 mL). The sodium carbonate (3.18 g, 30.0 mmol) in water (30 mL) was added into the solution and stirred for 24 h at 85 ℃. Upon cooling to room temperature, water (120 mL), dichloromethane (120 mL) was added and stirred. After filtration of the solution, the solution was partitioned between dichloromethane and water. The product was extracted from the organic layer. The aqueous layer was further extracted twice with dichloromethane (120 mL). The combined organic layer was dried over anhydrous Na_2SO_4 and evaporated under reduced pressure. The product was purified by column chromatography on silica gel (eluent: 5/1, v/v, dichloromethane : ethyl acetate) to give product M5 (4.48 g, 89%) as a pale yellow solid. m.p. 213-214; ^{1}H NMR (400 MHz, $CDCl_3$, 298 K) δ 8.87 (s, 2H), 8.70 (s, 1H), 8.11 (d, *J* = 8.0 Hz, 2H), 7.95-7.87 (m, 4H), 7.60 (s, 1H), 7.33 (d, *J* = 12.0 Hz, 2H), 6.64-6.60 (m, 4H), 3.87 (s, 6H), 3.84 (s, 6H). ^{13}C NMR (100 MHz, $CDCl_3$, 298 K) δ 161.4, 158.2, 155.3, 150.4, 140.2, 137.7, 133.0, 131.4, 129.6, 127.6, 125.7, 120.3, 120.1, 105.4, 99.5, 56.0, 55.9. HRMS (ESI) m/z: $[M+H]^+$ calcd for $[C_{32}H_{29}N_2O_4]^+$, 505.2122; found, 505.2130.

C7. To the solution of M7 (1.08 g, 2.0 mmol) in 1,2-dichloroethane (200 mL) was added paraformaldehyde (0.180 g, 6.00 mmol). Boron trifluoride diethyl etherate (0.260 ml, 2.00 mmol) was then added to the reaction mixture. The mixture was stirred at 25 °C for 5 hours. Then the reaction was quenched by addition of 150 mL saturated aqueous $NaHCO_3$. The solution was partitioned between dichloromethane and saturated aqueous $NaHCO_3$. The product was extracted from the organic layer. The aqueous layer was further extracted twice with dichloromethane (150 mL). The combined organic layer was dried over anhydrous Na_2SO_4 and concentrated. The product was purified by column chromatography on silica gel (eluent : dichloromethane: ethyl acetate = 1:1) to obtain product C7 (0.248 g, 24%) as a white solid. m.p. >320°C; ^{1}H NMR (400 MHz, $CDCl_3$, 298 K) δ 8.81 (s, 4H), 8.66 (s, 2H), 8.07 (d, *J* = 8.0 Hz, 4H), 7.90-7.86 (m, 8H), 7.58 (s, 2H), 6.99 (s, 4H), 6.59 (s, 4H), 3.93 (s, 4H), 3.90 (s, 12H), 3.84 (s, 12H). ^{13}C NMR (100 MHz, $CDCl_3$, 298 K) δ 158.6, 156.3, 154.9, 150.2, 140.0, 137.5, 133.0, 131.7, 129.2, 127.1, 125.7, 121.8, 120.2, 120.0, 119.1, 105.3, 99.3, 96.1, 56.0, 55.4, 27.6. HRMS (ESI) m/z: $[M+H]^+$ calcd for $[C_{66}H_{57}N_4O_8]^+$, 1033.4171; found, 1033.4171.

1.2 ^{1}H NMR, ^{13}C NMR, HMRS spectra

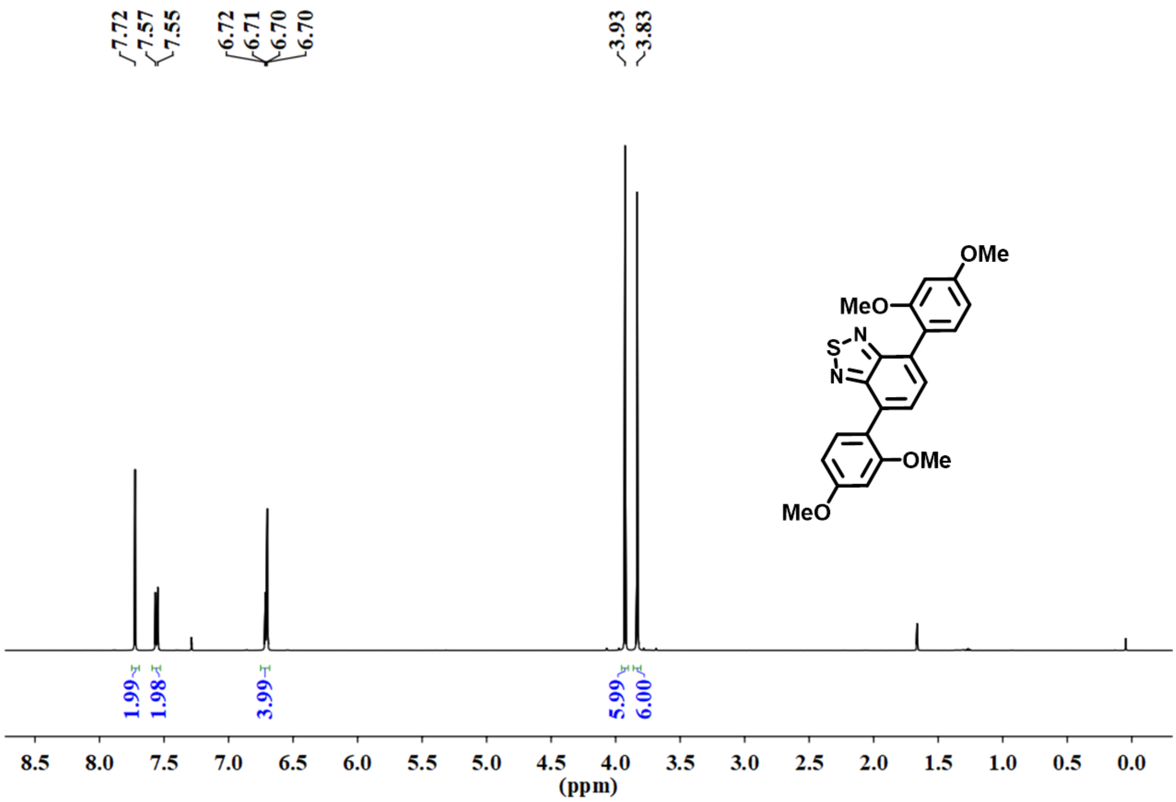

Supplementary Figure 2. ^{1}H NMR spectrum (500 MHz, $CDCl_3$, 298 K) of BT-M.

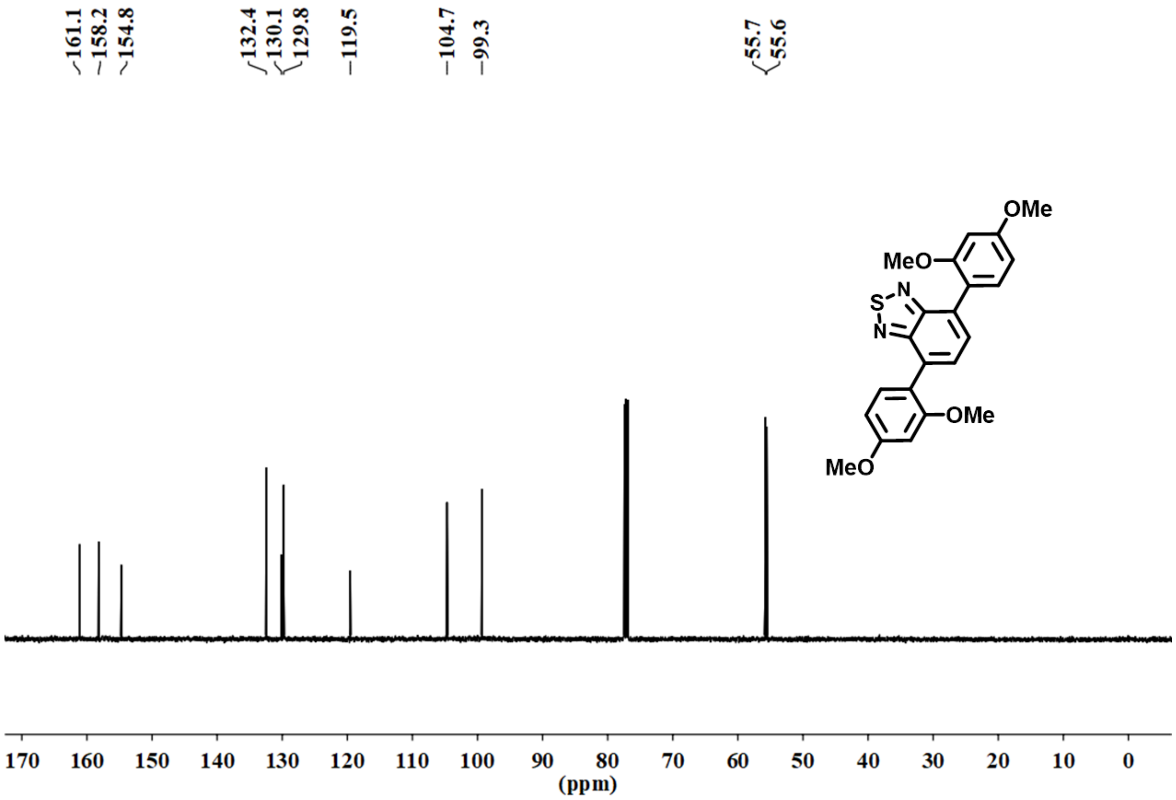

Supplementary Figure 3. ^{13}C NMR spectrum (125 MHz, $CDCl_3$, 298 K) of BT-M.

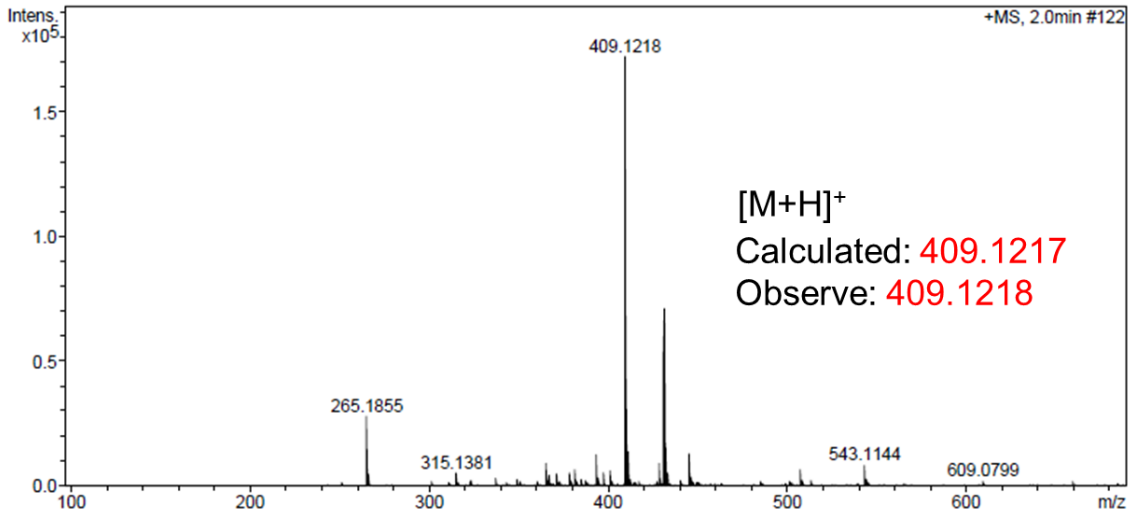

Supplementary Figure 4. HRMS spectrum of BT-M.

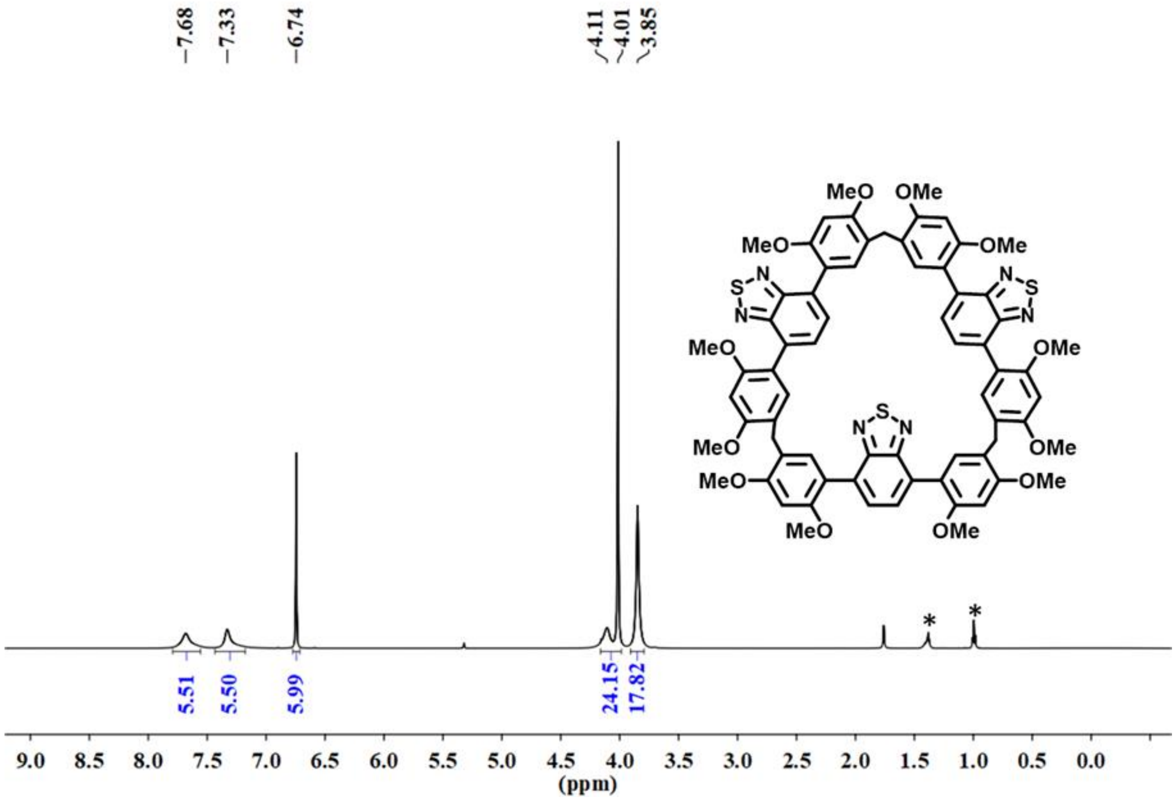

Supplementary Figure 5. ^{1}H NMR spectrum (500 MHz, CD_2Cl_2, 298 K) of BT-LC. (* = petroleum ether peak signals)

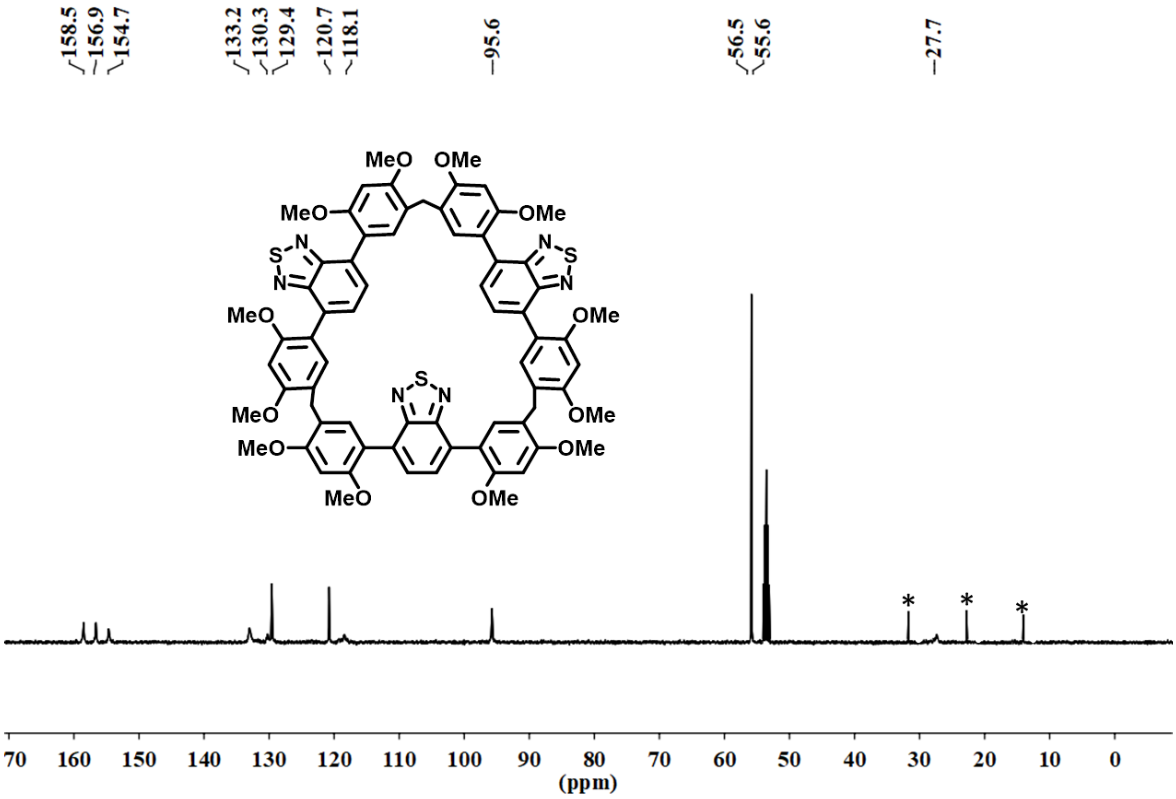

Supplementary Figure 6. ^{13}C NMR spectrum (125 MHz, CD_2Cl_2, 298 K) of BT-LC. (* = petroleum ether peak signals)

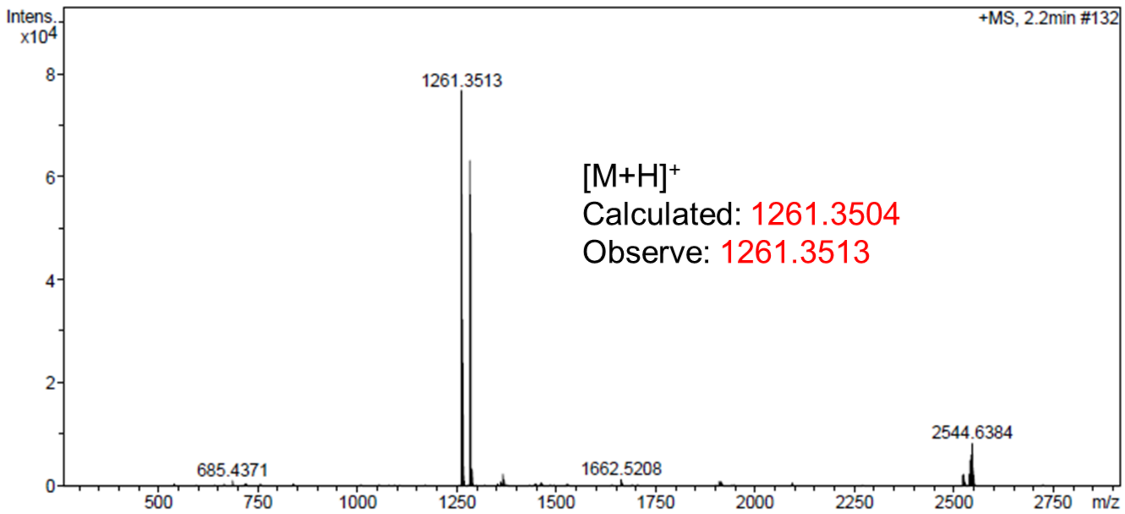

Supplementary Figure 7. HMRS spectrum of BT-LC.

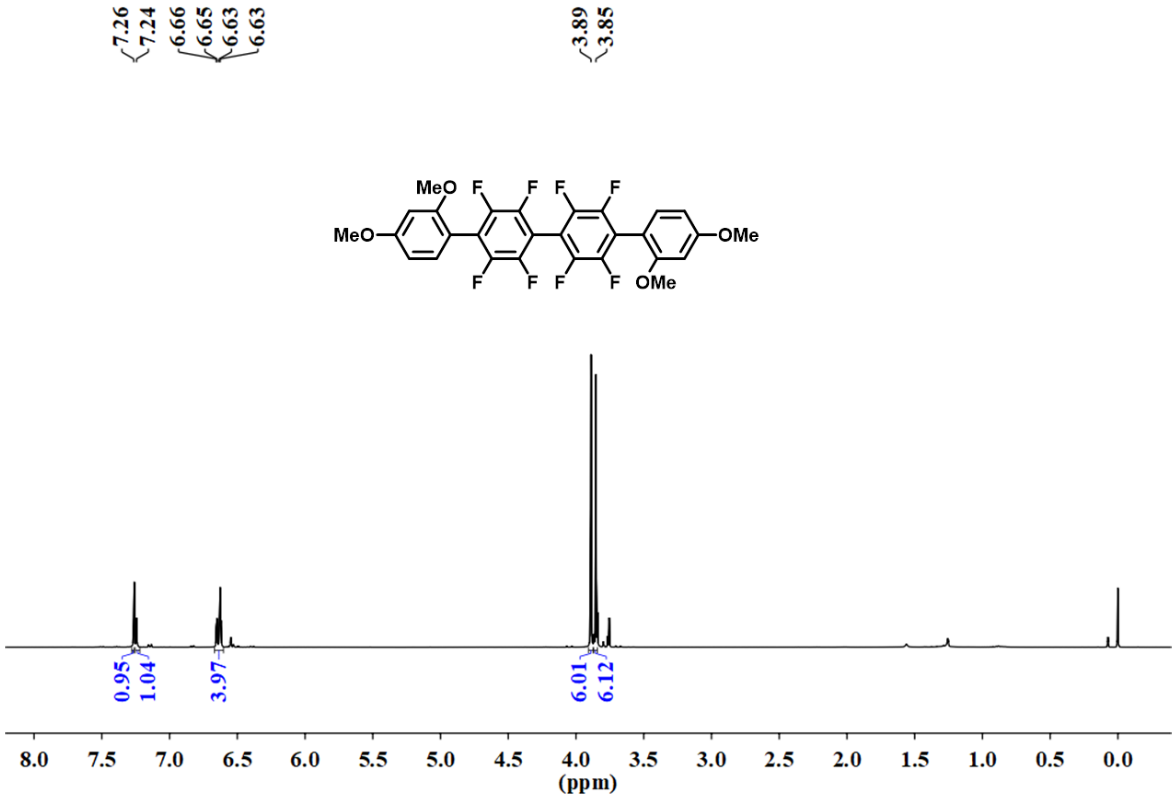

Supplementary Figure 8. ^{1}H NMR spectrum (400 MHz, $CDCl_3$, 298 K) of M4.

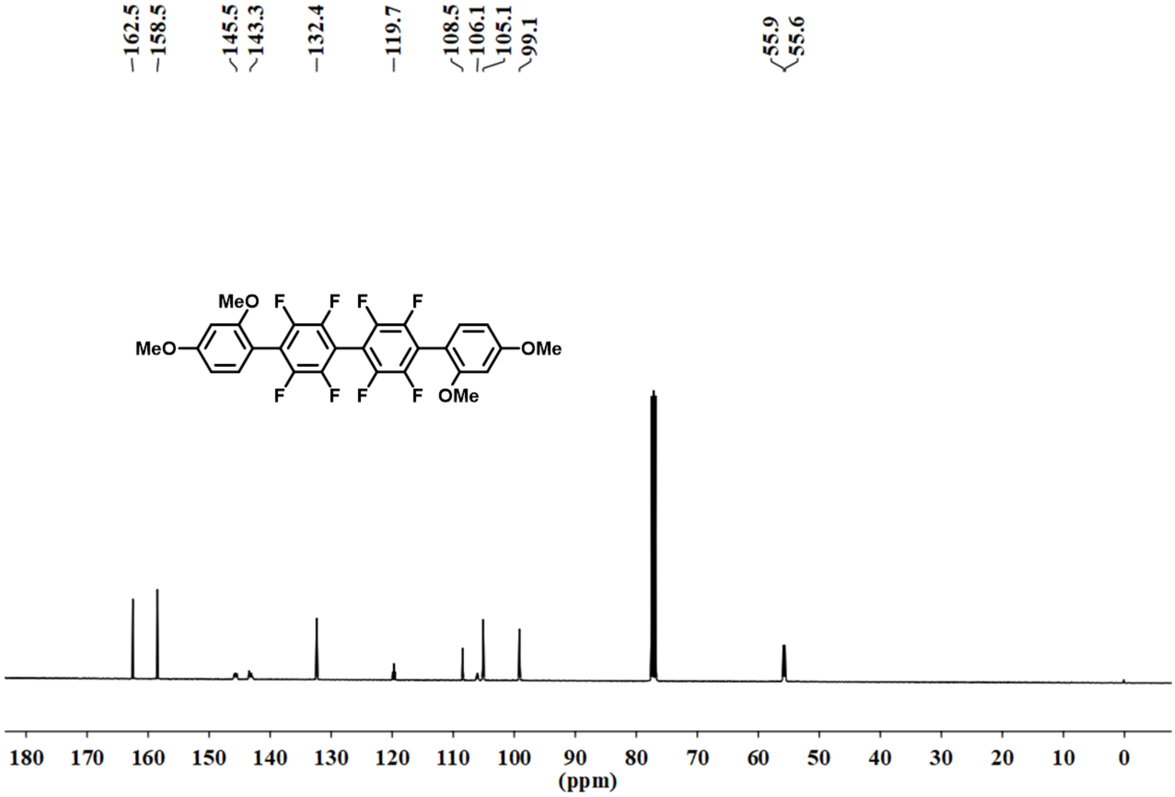

Supplementary Figure 9. ^{13}C NMR spectrum (100 MHz, $CDCl_3$, 298 K) of M4.

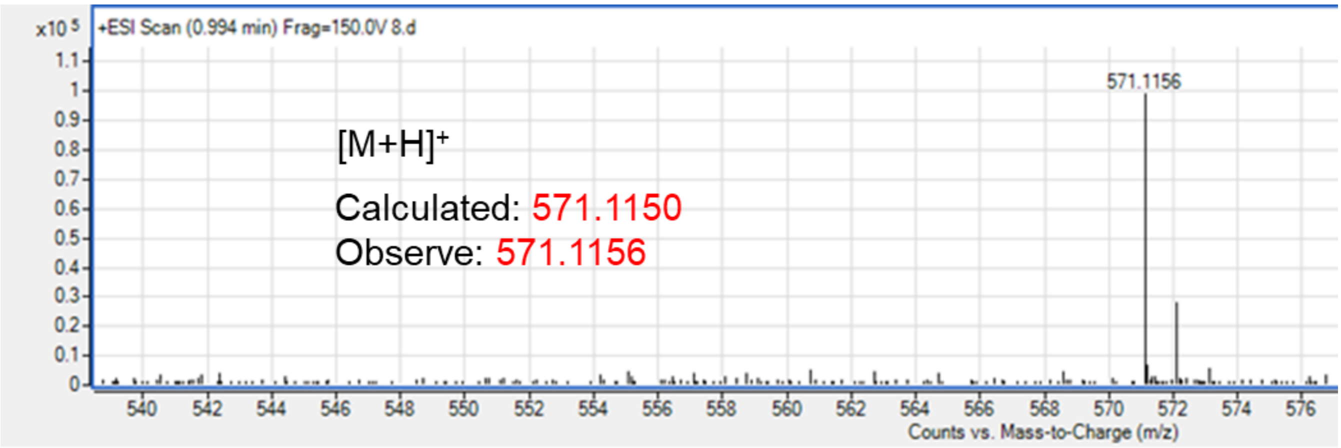

Supplementary Figure 10. HMRS spectrum of M4.

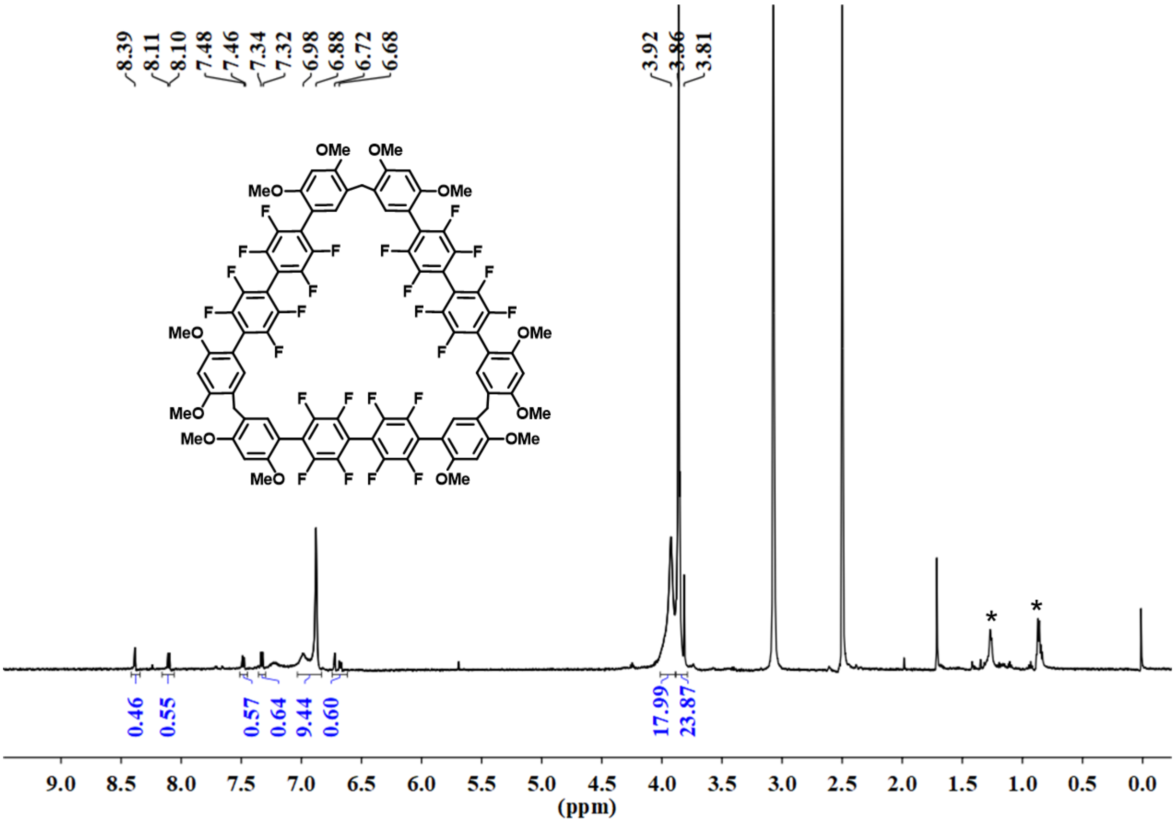

Supplementary Figure 11. ^{1}H NMR spectrum (400 MHz, DMSO, 353 K) of C4.

Due to the isomerization, the protons of C4 are complicated even at high temperature of 353K. It was confirmed by the HRMS (Supplementary Figure 13). We also got its X-ray crystal structure.

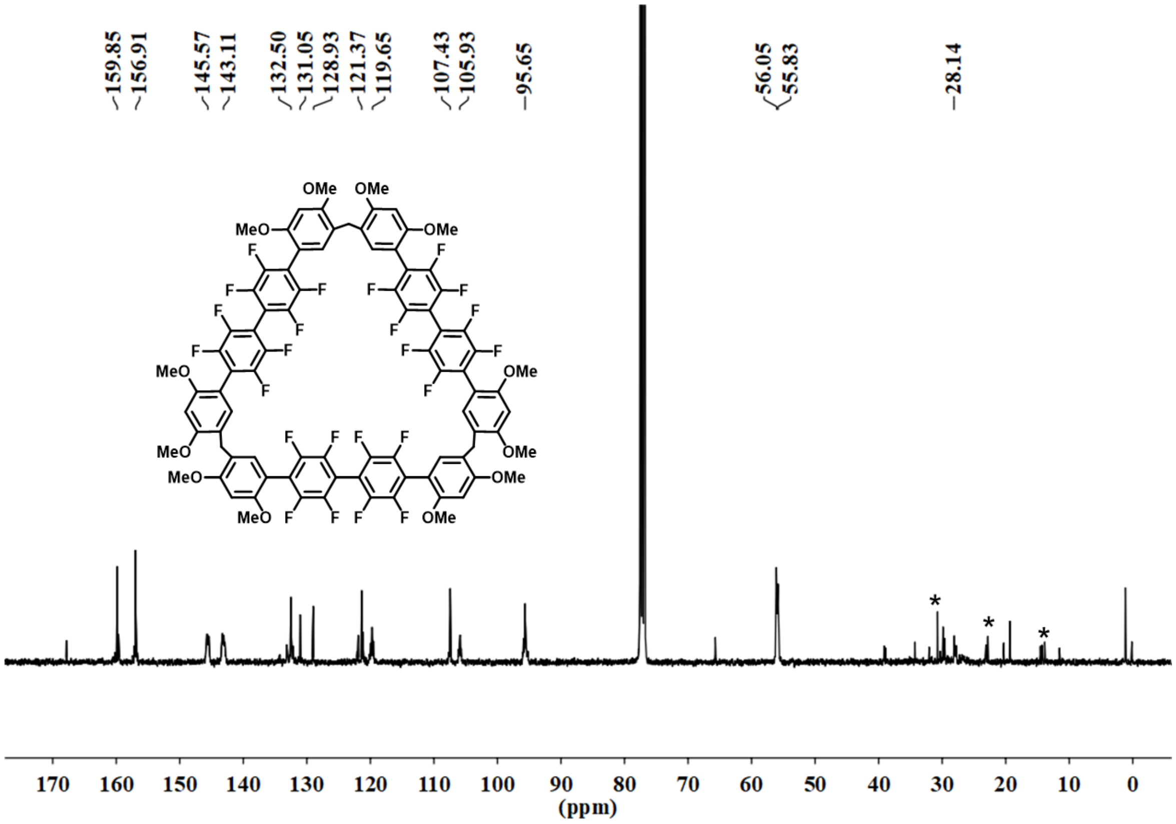

Supplementary Figure 12. ^{13}C NMR spectrum (100 MHz, $CDCl_3$, 298 K) of C4.

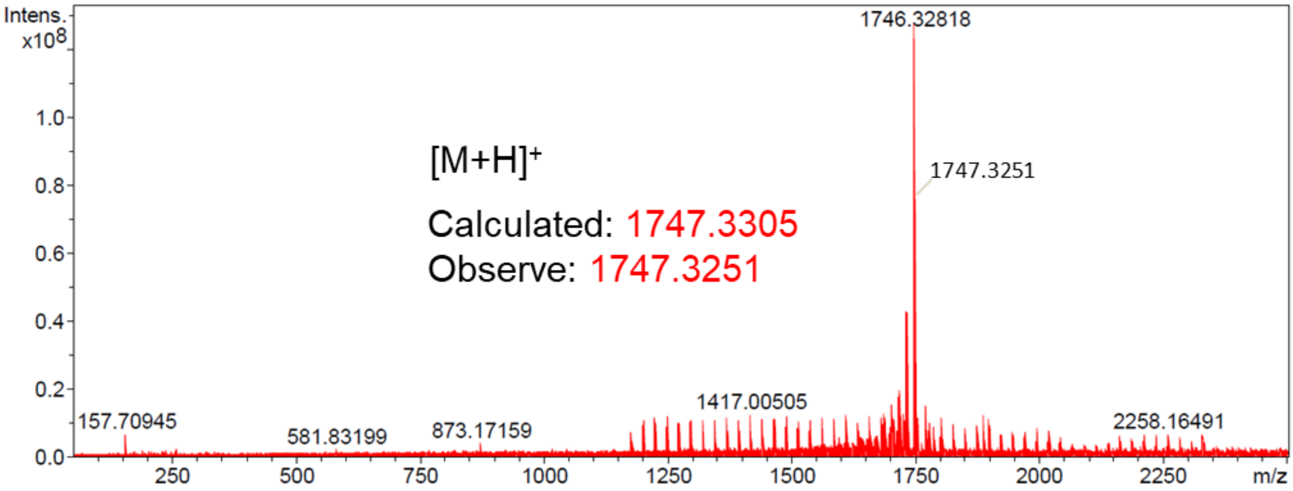

Supplementary Figure 13. HMRS spectrum of C4.

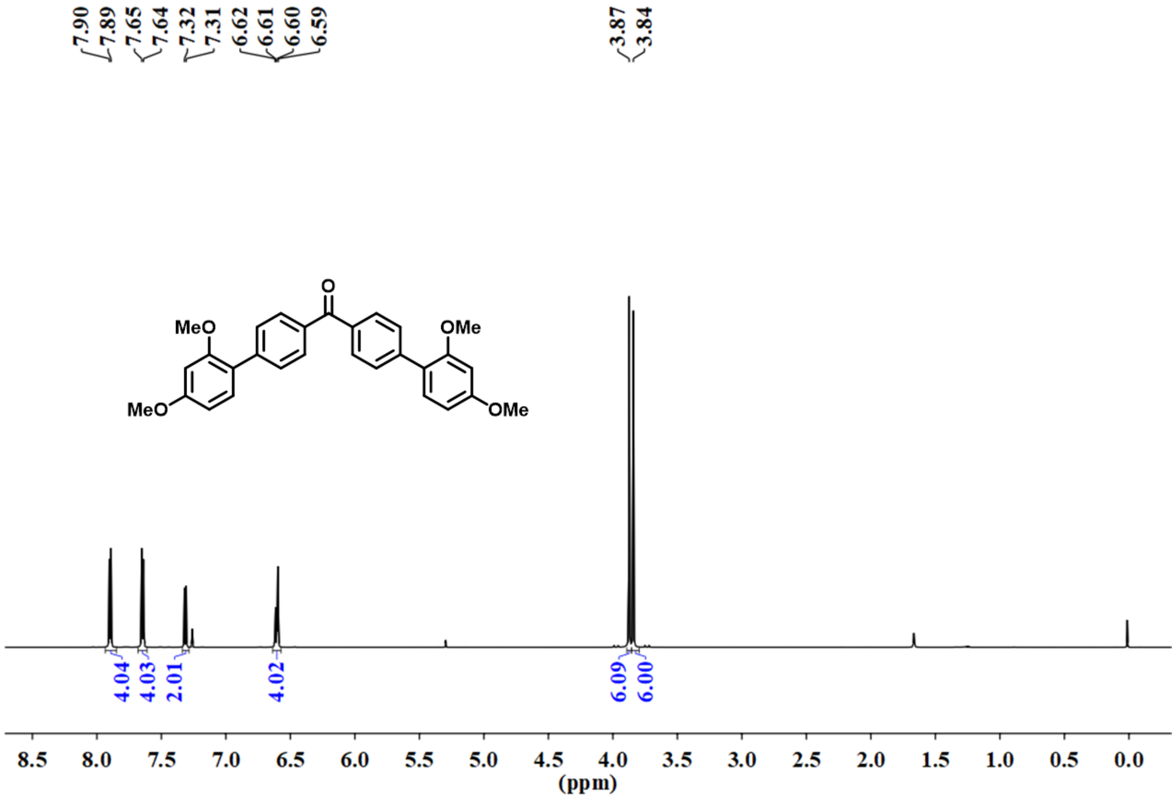

Supplementary Figure 14. ^{1}H NMR spectrum (600 MHz, $CDCl_3$, 298 K) of M5.

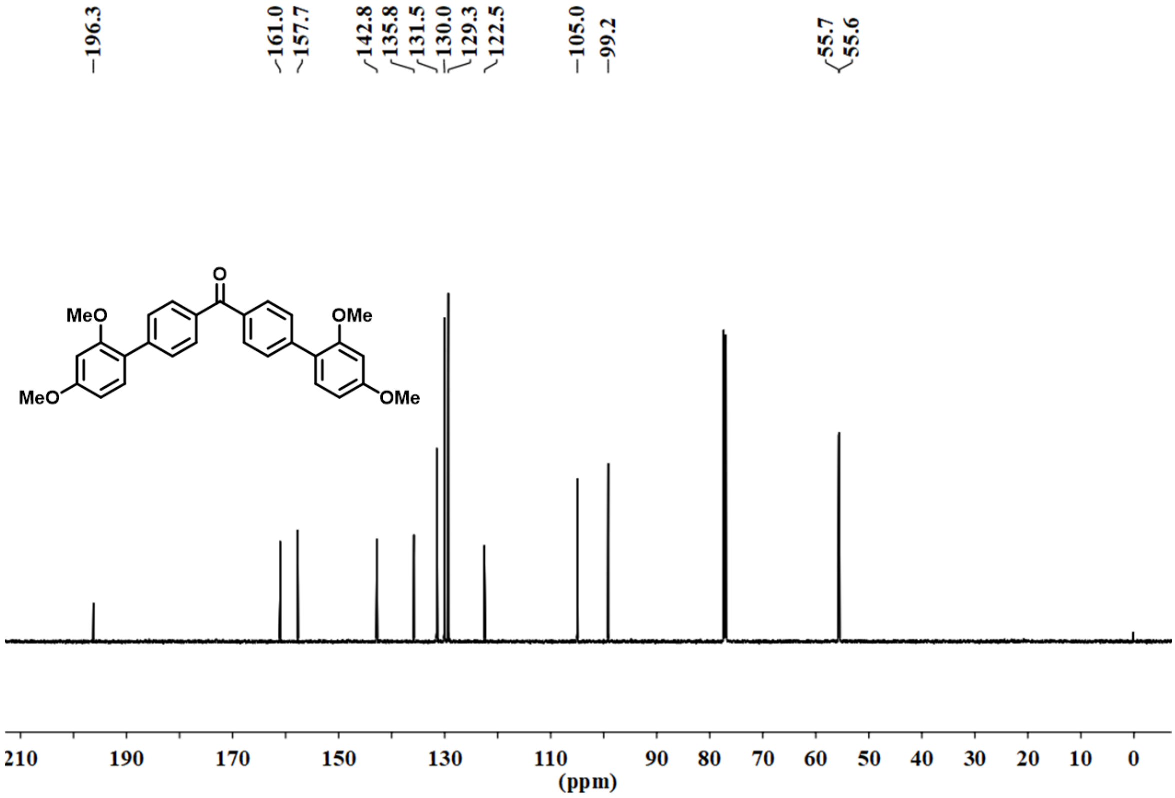

Supplementary Figure 15. ^{13}C NMR spectrum (150 MHz, $CDCl_3$, 298 K) of M5.

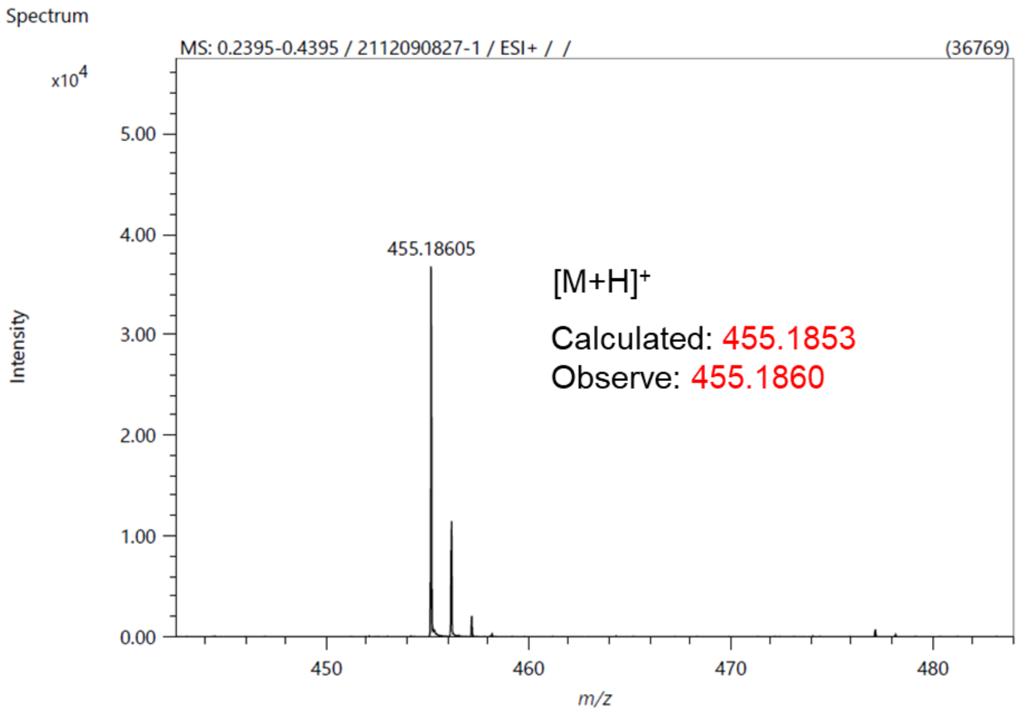

Supplementary Figure 16. HMRS spectrum of M5.

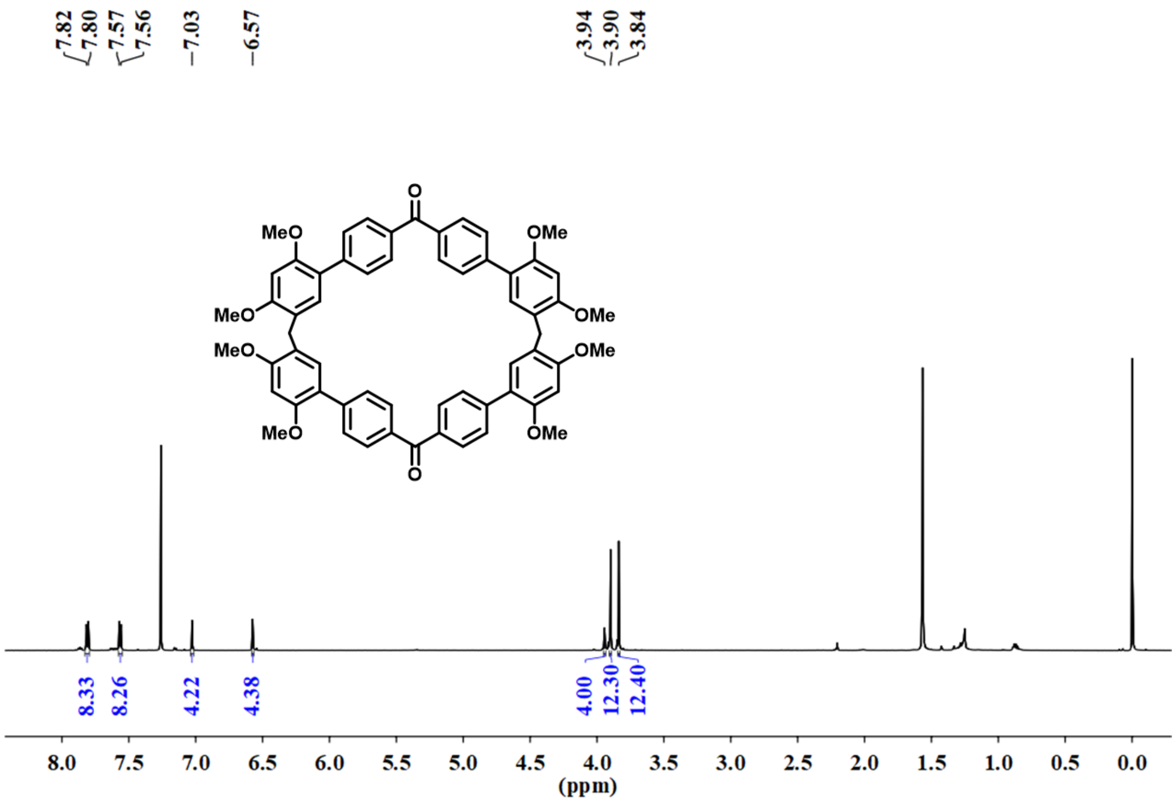

Supplementary Figure 17. ^{1}H NMR spectrum (600 MHz, $CDCl_3$, 298 K) of C5.

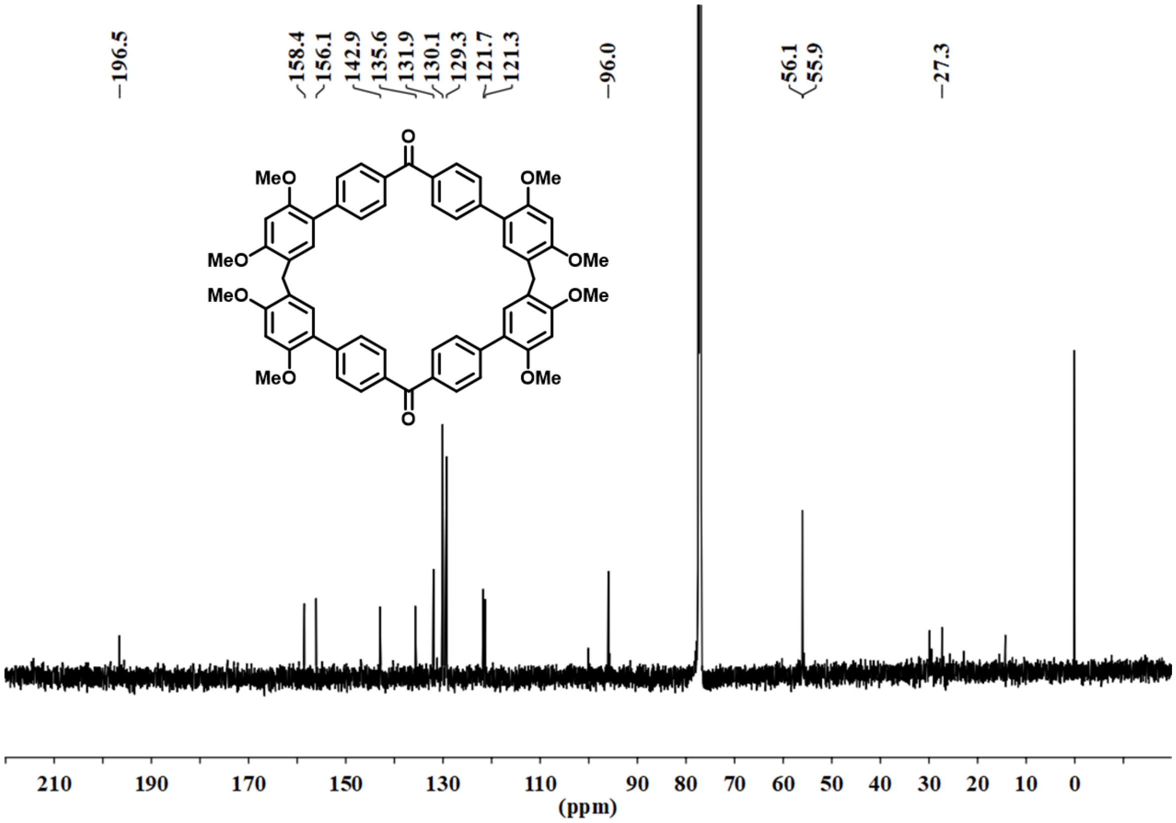

Supplementary Figure 18. ^{13}C NMR spectrum (150 MHz, $CDCl_3$, 298 K) of C5.

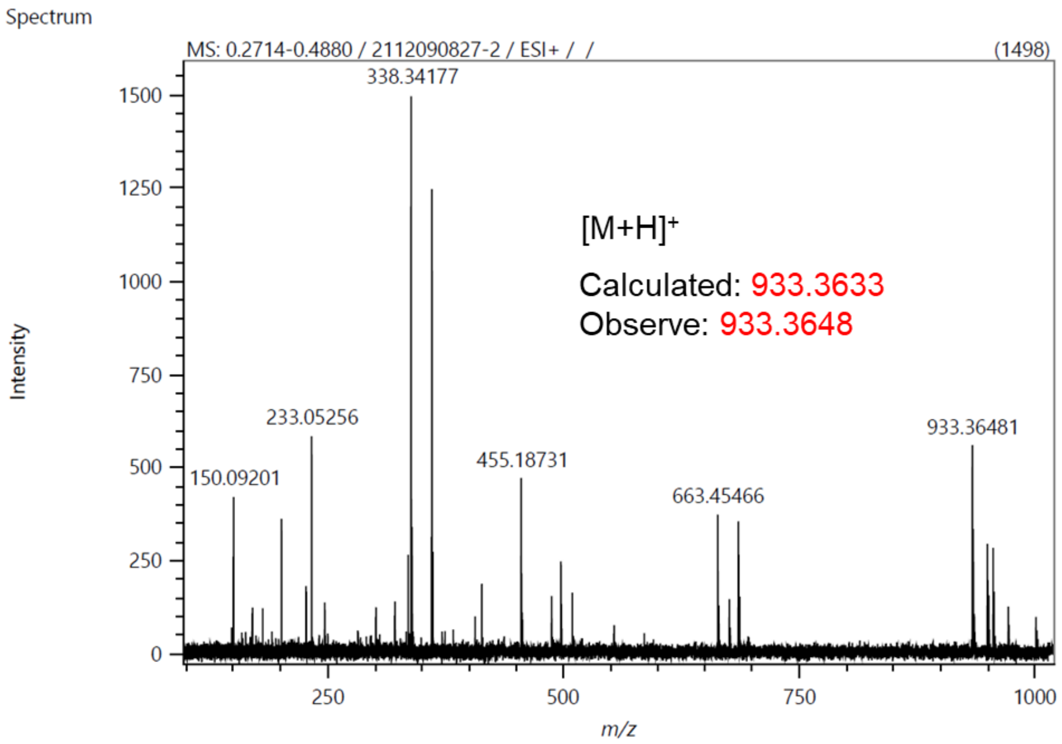

Supplementary Figure 19. HMRS spectrum of C5.

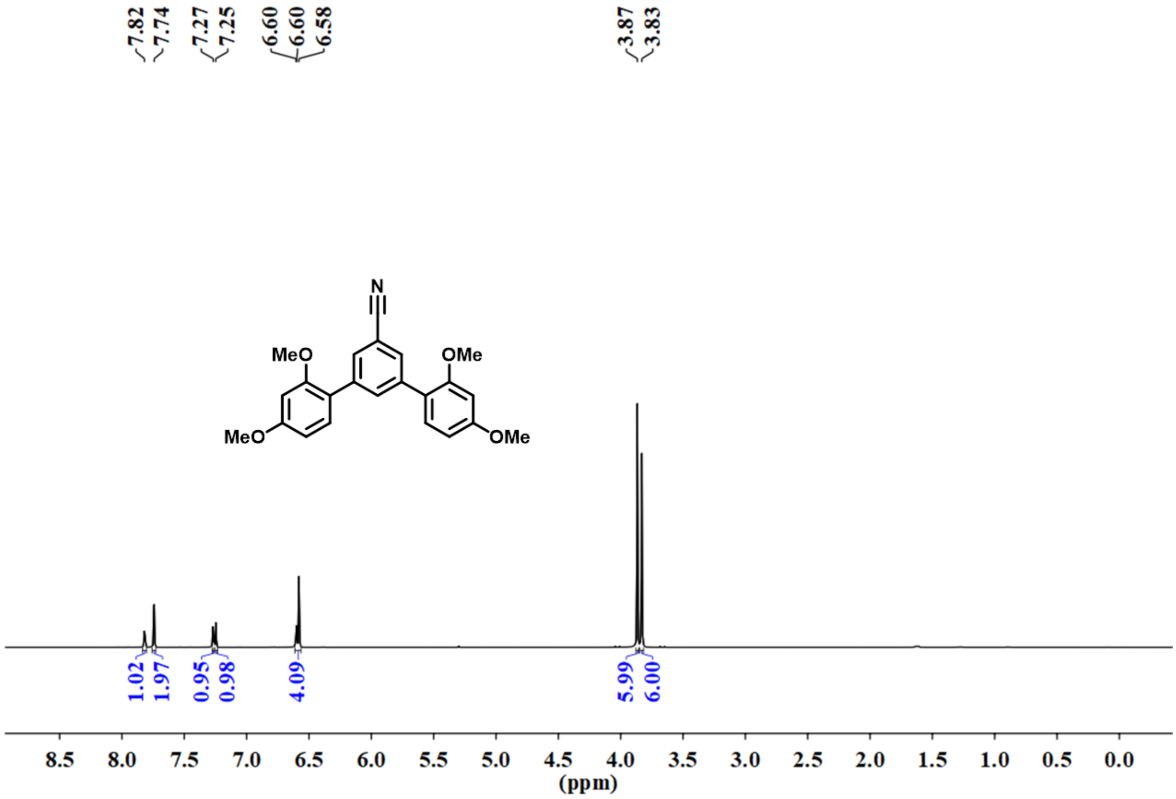

Supplementary Figure 20. ^{1}H NMR spectrum (400 MHz, $CDCl_3$, 298 K) of M6.

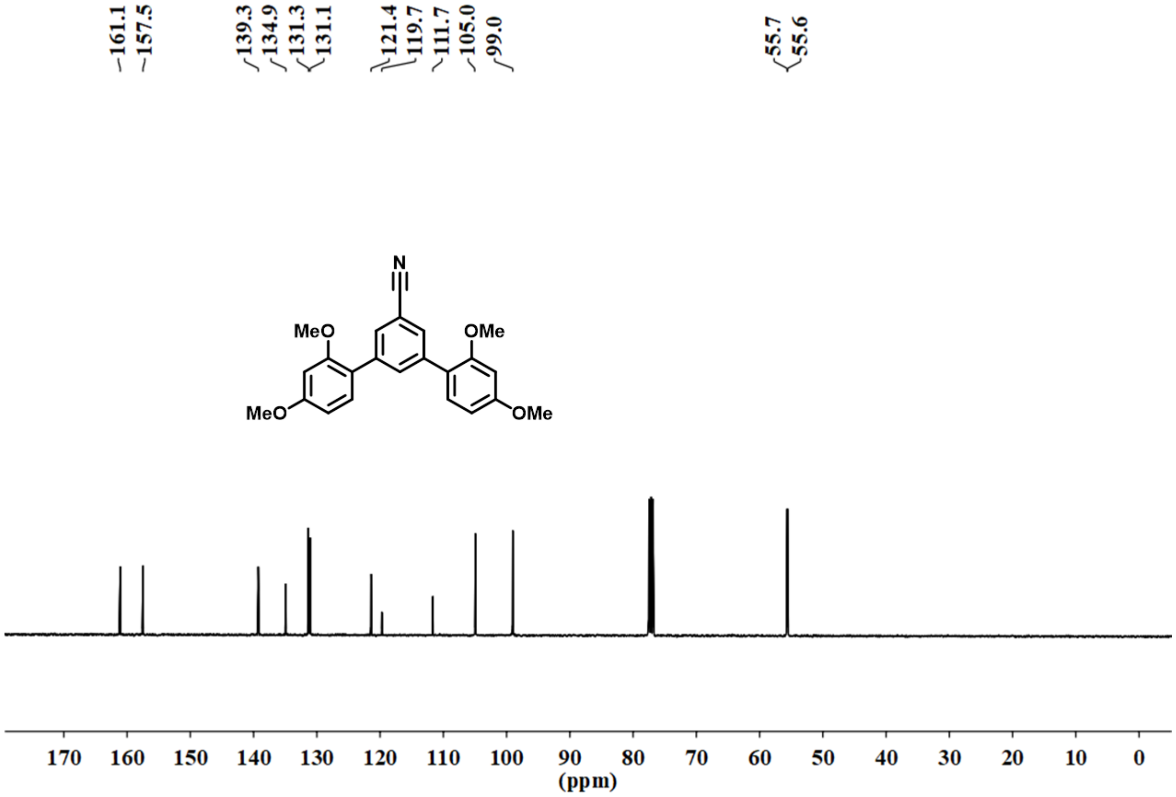

Supplementary Figure 21. ^{13}C NMR spectrum (100 MHz, $CDCl_3$, 298 K) of M6.

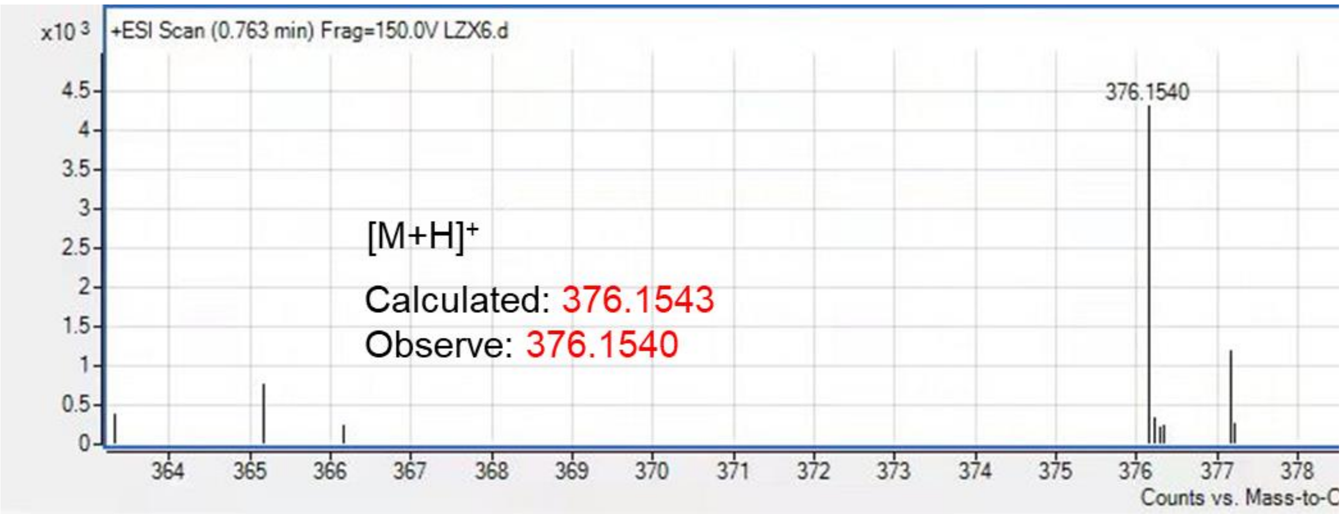

Supplementary Figure 22. HMRS spectrum of M6.

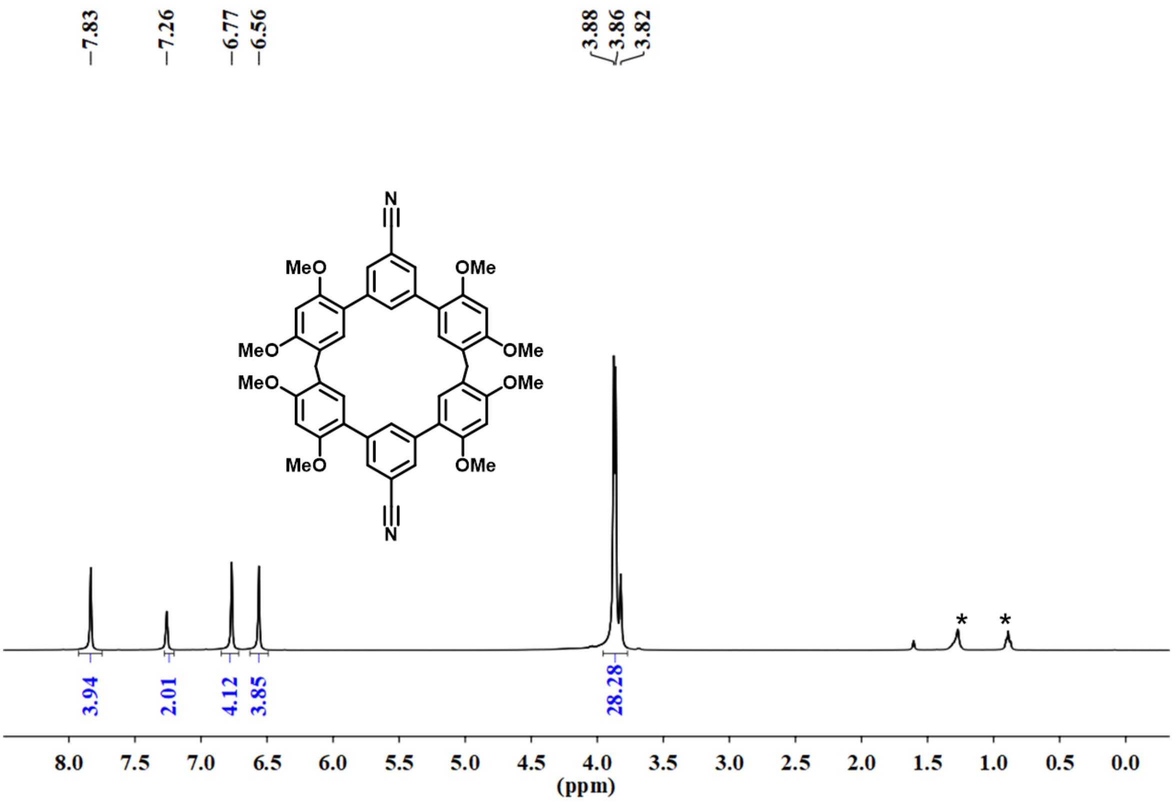

Supplementary Figure 23. ^{1}H NMR spectrum (400 MHz, $CDCl_3$, 298 K) of C6 (* = petroleum ether peak signals).

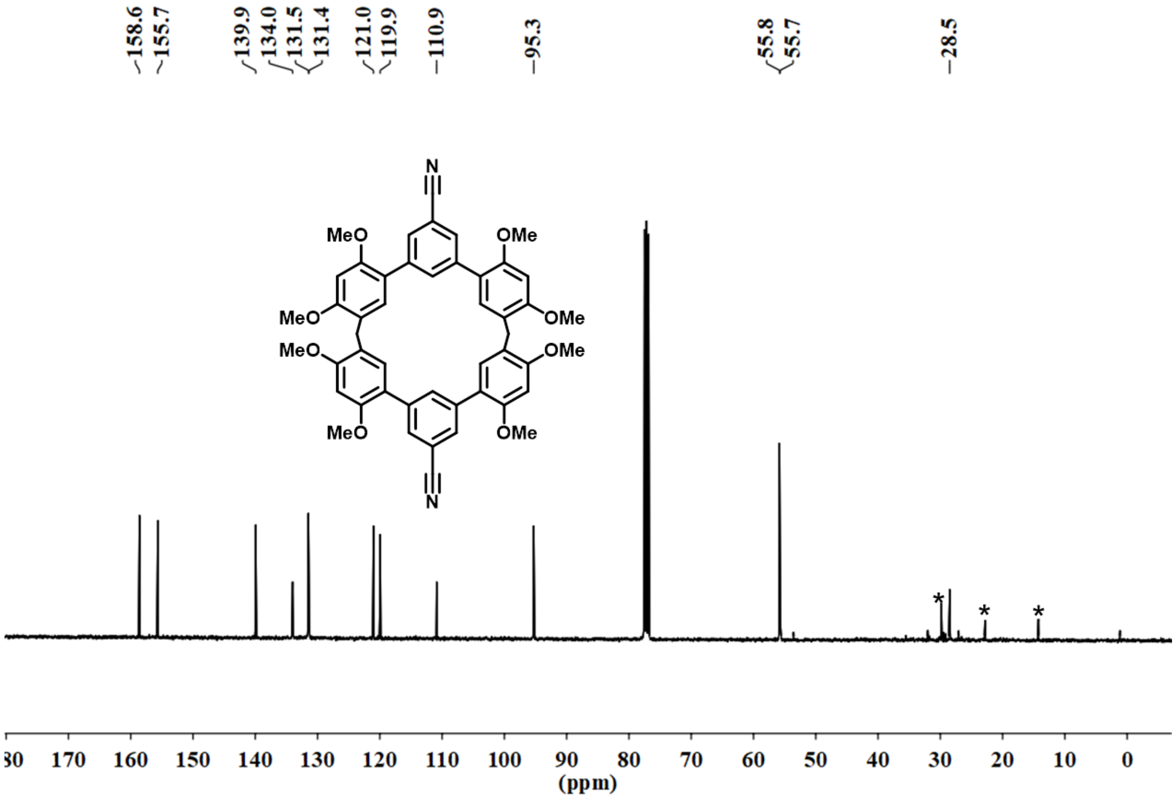

Supplementary Figure 24. ^{13}C NMR spectrum (100 MHz, $CDCl_3$, 298 K) of C6 (* = petroleum ether peak signals).

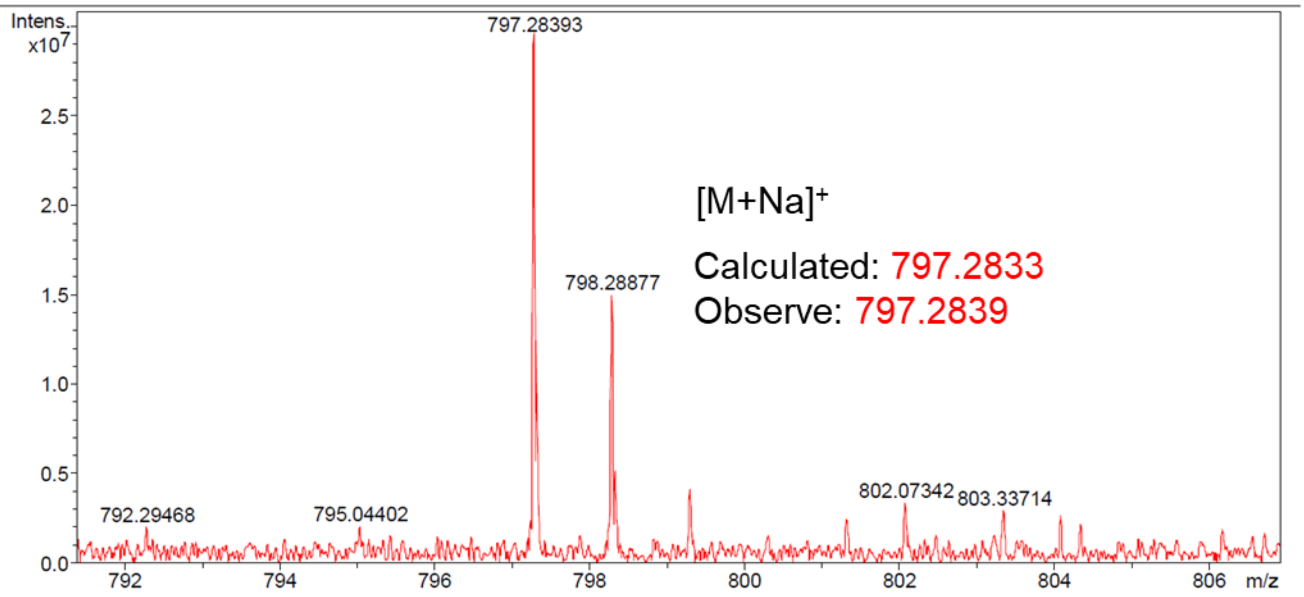

Supplementary Figure 25. HMRS spectrum of C6.

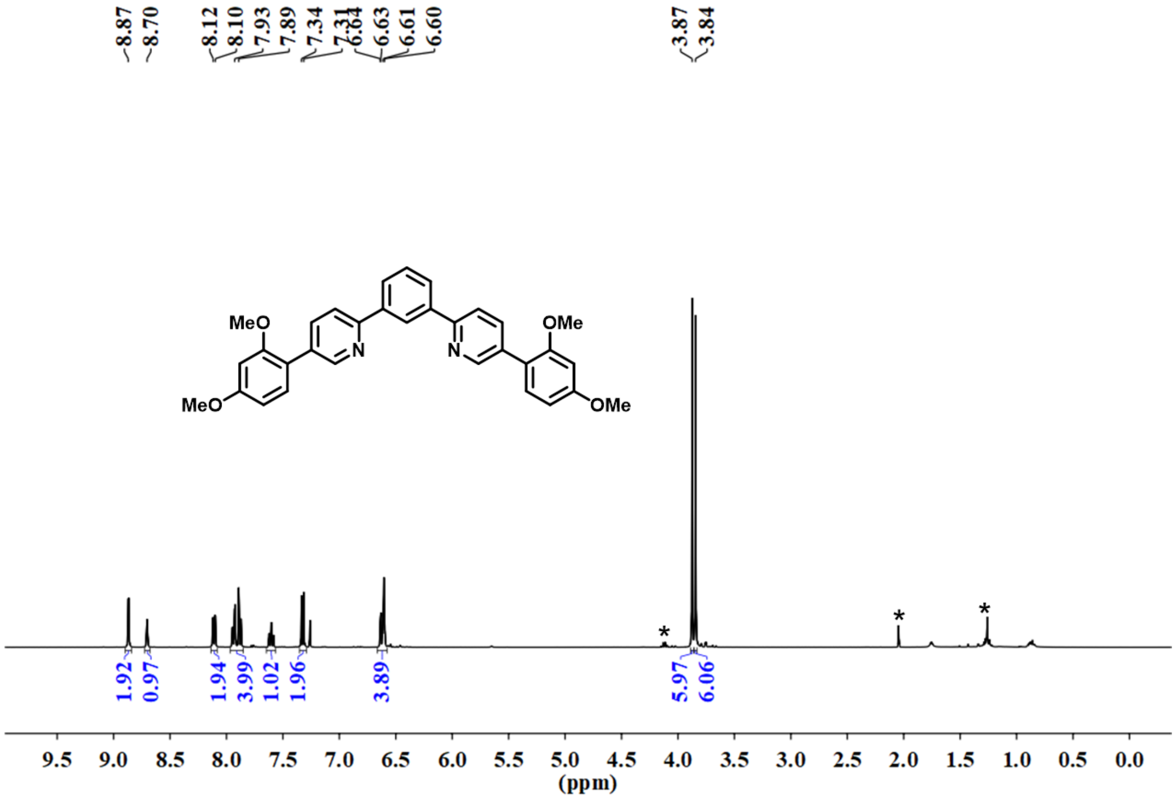

Supplementary Figure 26. ^{1}H NMR spectrum (400 MHz, $CDCl_3$, 298 K) of M7 (* = ethyl acetate peak signals).

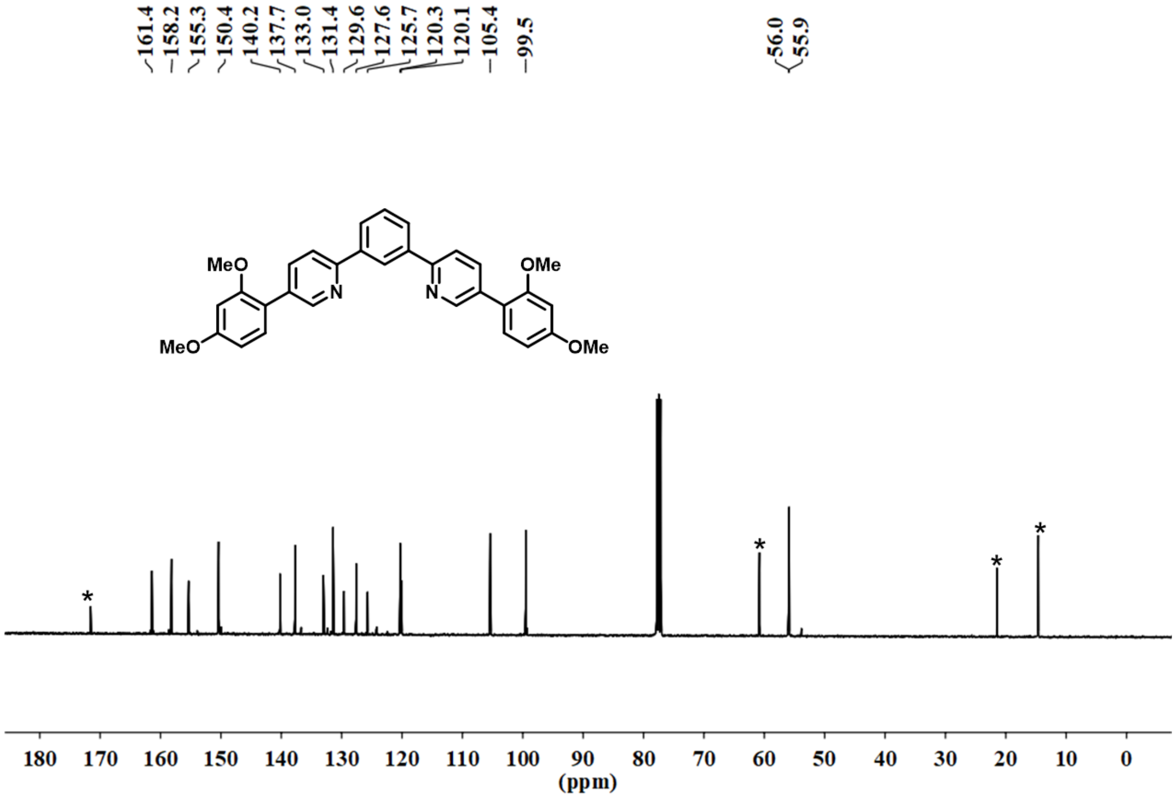

Supplementary Figure 27. ^{13}C NMR spectrum (100 MHz, $CDCl_3$, 298 K) of M7 (* = ethyl acetate peak signals).

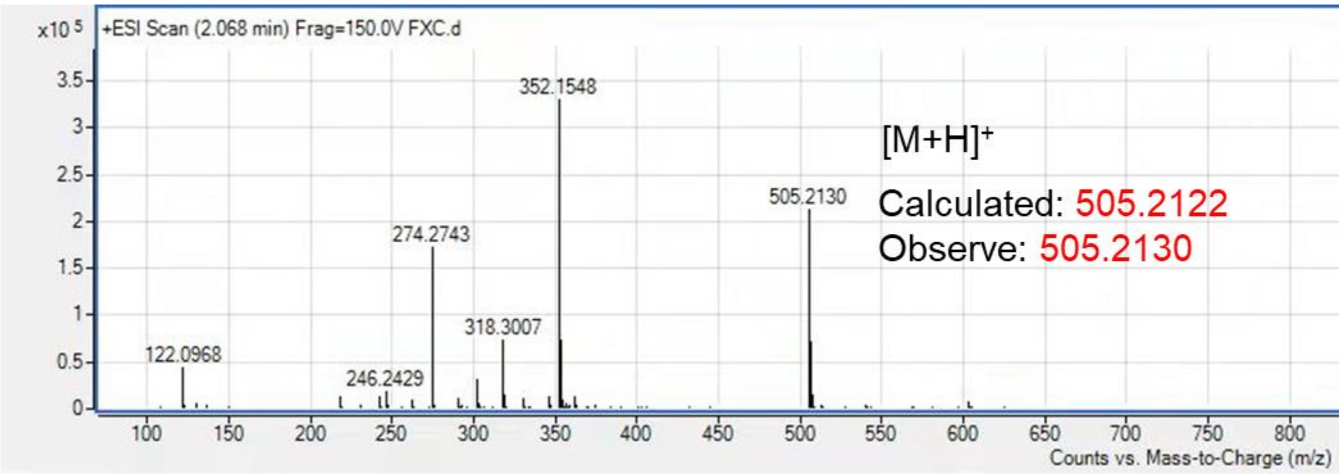

Supplementary Figure 28. HMRS spectrum of M7.

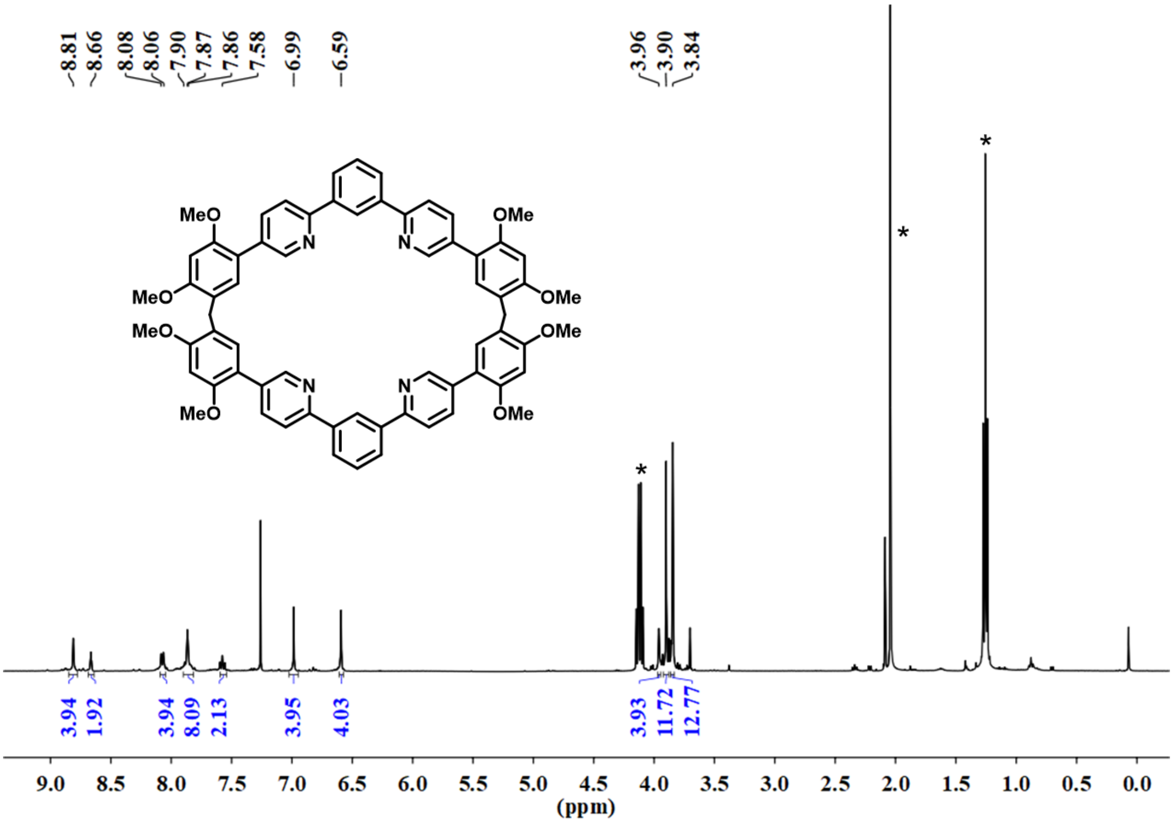

Supplementary Figure 29. ^{1}H NMR spectrum (400 MHz, $CDCl_3$, 298 K) of C7 (* = ethyl acetate peak signals).

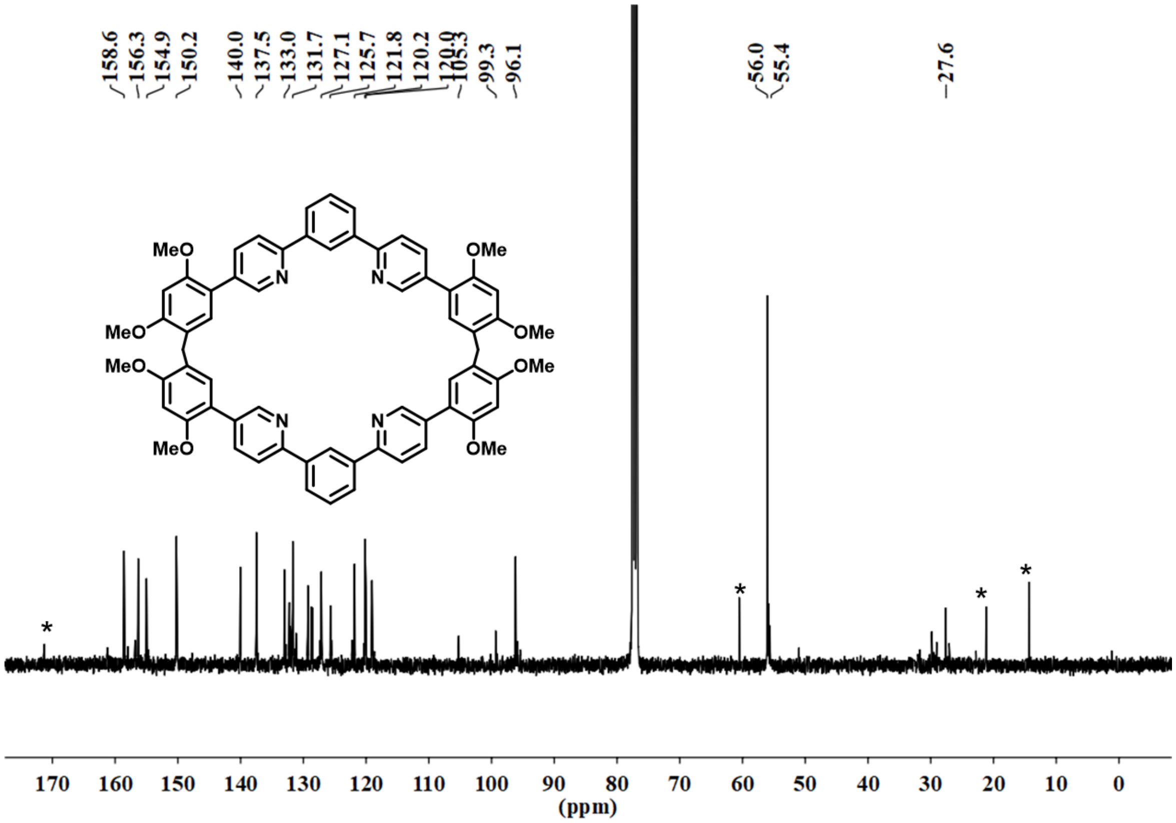

Supplementary Figure 30. ^{13}C NMR spectrum (100 MHz, $CDCl_3$, 298 K) of C7 (* = ethyl acetate peak signals).

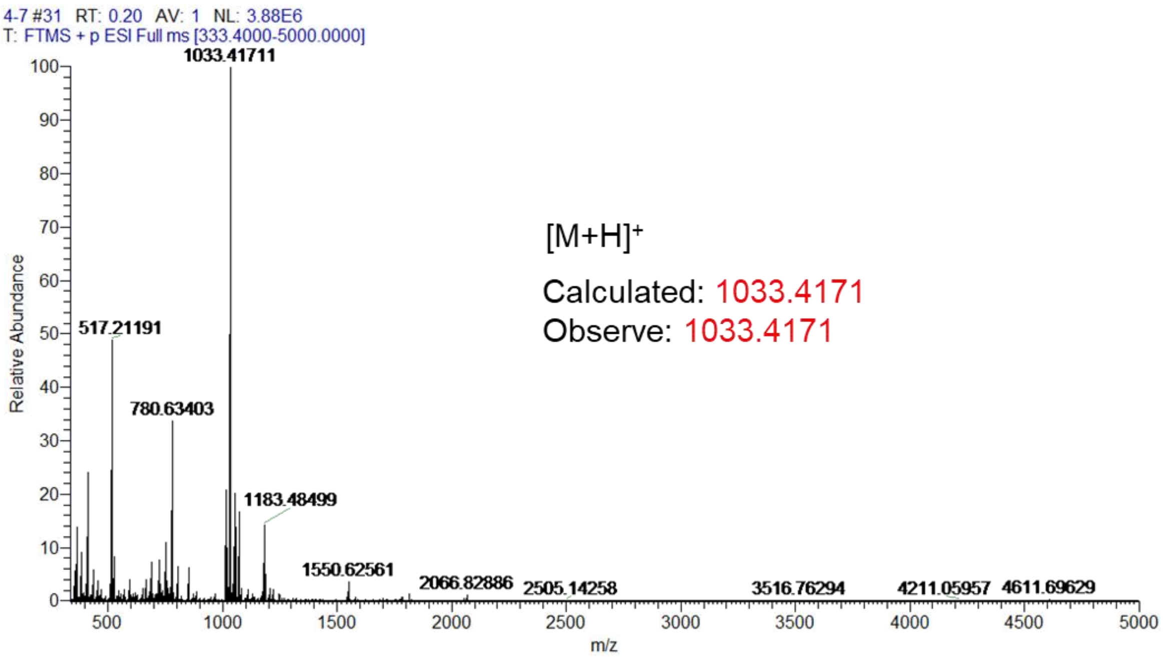

Supplementary Figure 31. HMRS spectrum of C7.

2. Supplementary Discussion

2.1 Photophysical properties

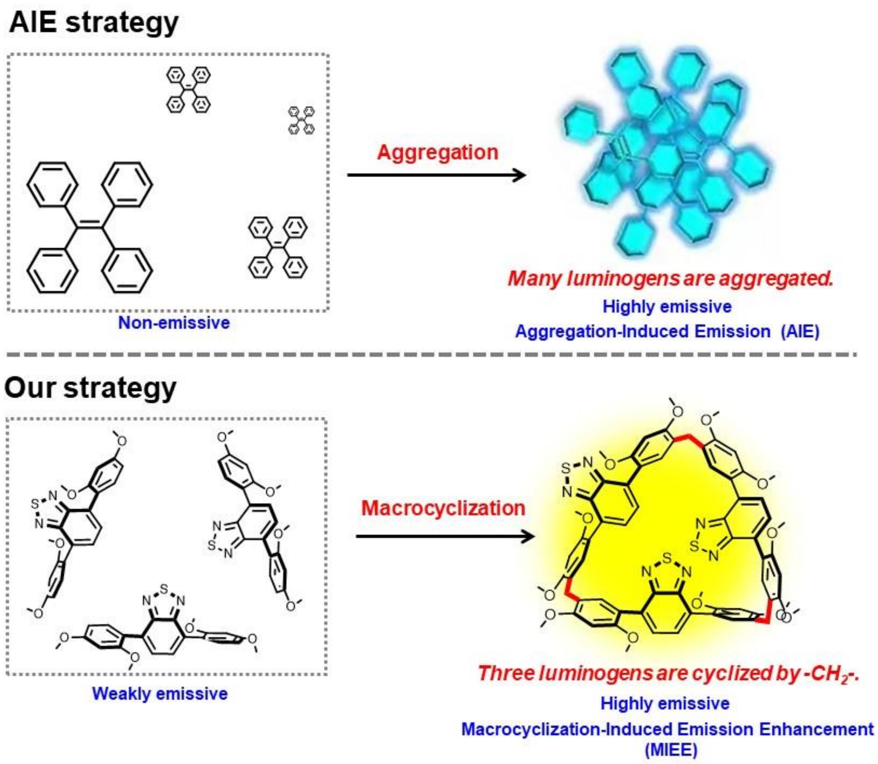

Supplementary Scheme 1. Illustration of aggregation-induced emission (AIE) and macrocyclization-induced emission enhancement (MIEE).[3]

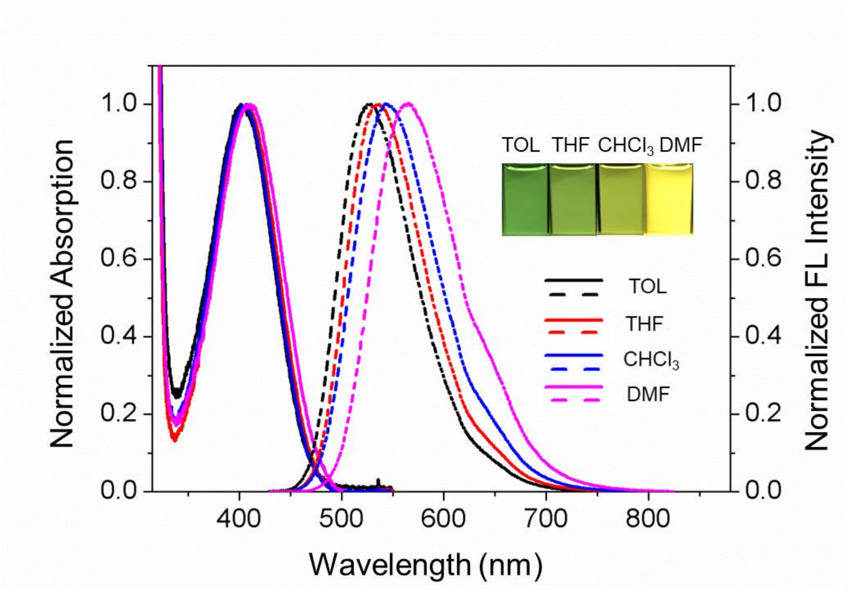

Supplementary Figure 32. Normalized UV/Vis absorption spectra (solid lines) and fluorescence spectra (dashed lines) of BT-LC in different solvents (3.3×10^{-6} M; λ_{ex}=418 nm). Insets: photographs of BT-LC in different solvents under 365 nm UV illuminations.

Supplementary Table 1. Photophysical properties of BT-LC

	BT-LC		
	λ_{abs}(nm)	λ_{em}(nm)	Φ_{PL}(%)
TOL	401	529	86
THF	407	537	86
$CHCl_3$	406	545	83
DMF	410	561	89
Solid	-	562	99

λ_{abs} (nm): absorption maximum. λ_{em} (nm): fluorescence maximum. Φ_{PL}: absolute PL quantum yield (λ_{ex} = 418 nm).

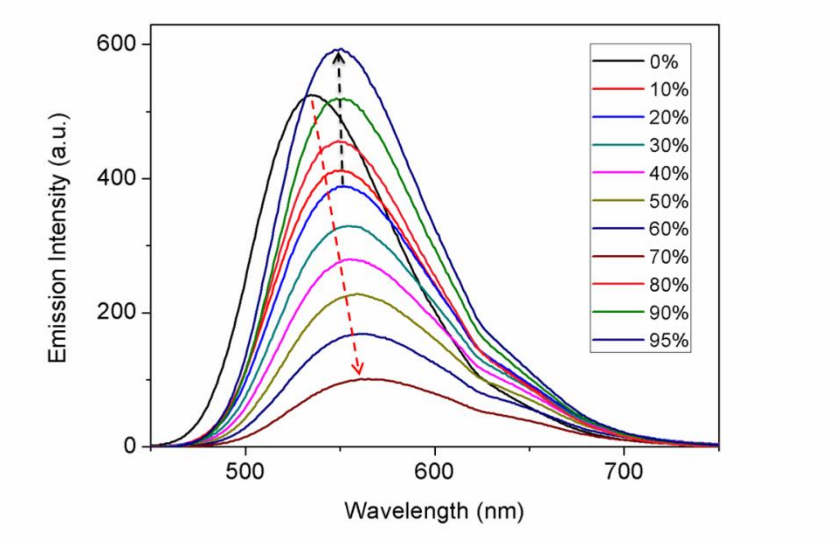

Supplementary Figure 33. Fluorescence spectra of BT-LC in THF/water mixtures with different fractions of water (0-95%, v/v).

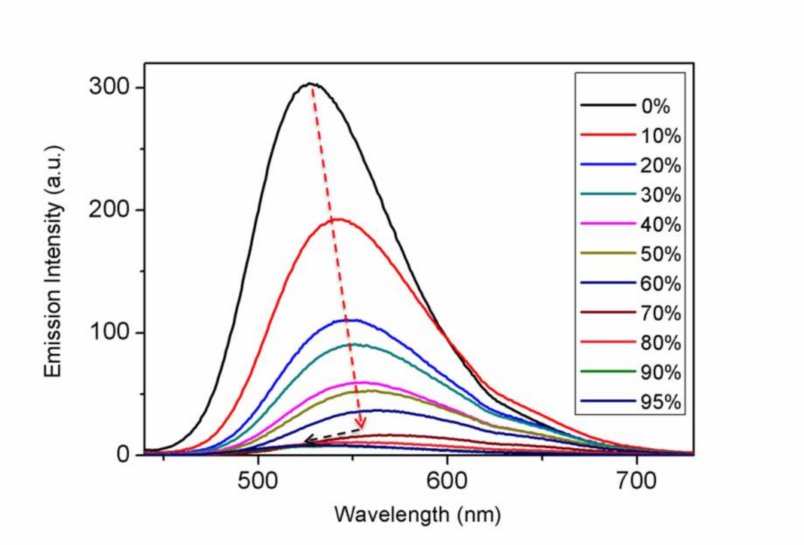

Supplementary Figure 34. Fluorescence spectra of BT-M in THF/water mixtures with different fractions of water (0-95%, v/v).

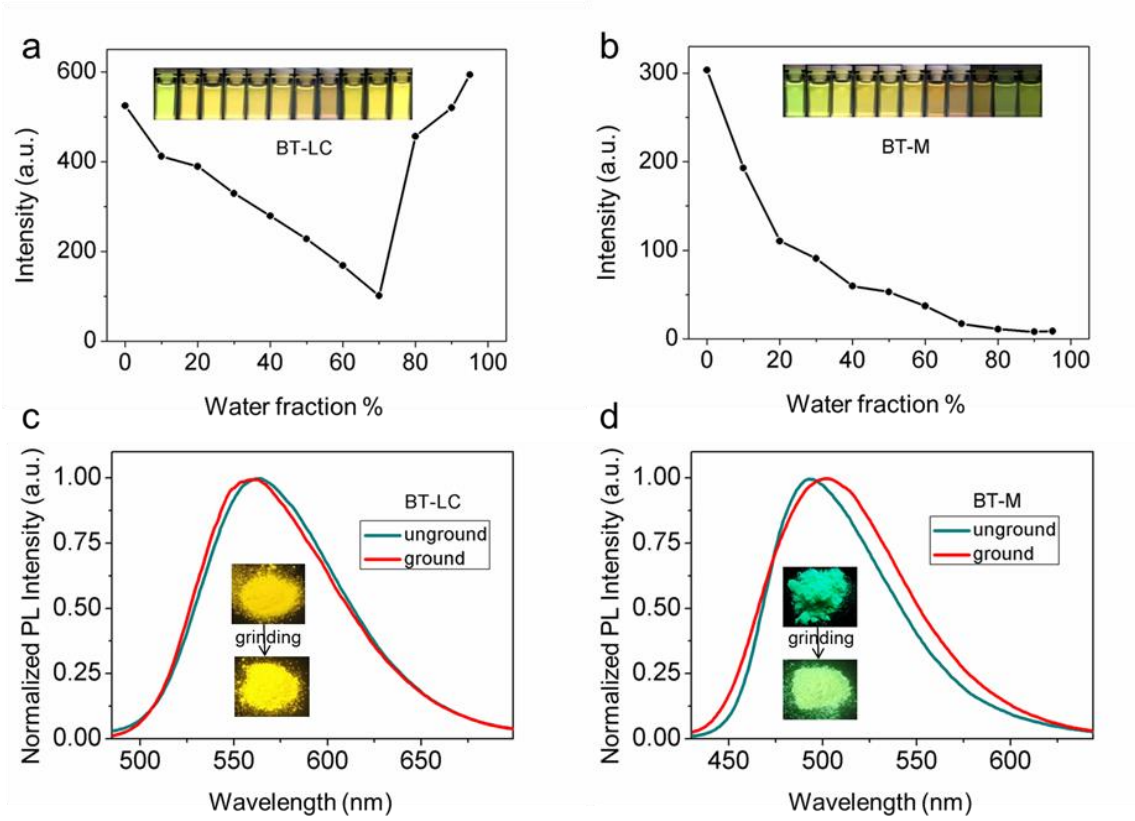

Supplementary Figure 35. Photo-physical behaviors of BT-LC and BT-M in aggregated state. **a** Fluorescence intensity of BT-LC and **b** Fluorescence intensity of BT-M in THF/water mixtures with different fractions of water (0-95%, v/v). Inset: photographs of the compounds with different f_w taken under 365 UV illumination. **c** Normalized fluorescence spectra of BT-LC and **d** Normalized fluorescence spectra of BT-M before and after grinding. Insets: photographs in unground and ground states under 365 nm UV illuminations.

From Supplementary Figure 35a and Supplementary Figure 33, the fluorescence intensity of BT-LC in THF/water mixtures becomes gradually reduced when the water fractions (f_w) increase from 0 to 70%. Meanwhile, the emission peak shows a visible red-shift from 535 to 565 nm. This emissive phenomenon is attributed to the TICT effect.[4-6] As the water fractions (f_w) further increases from 70 to 95%, the fluorescence intensity sharply recovers, exhibiting a typical AIE property, and the emission peak blue-shifts to 547 nm. Such photo-physical behaviors demonstrate that BT-LC can be viewed as a dual-state emission (DSE) molecule.[7-10] In sharp contrast, the emission intensity of the monomer BT-M swiftly decreases with increasing the f_w values, indicating a typical ACQ phenomenon (Supplementary Figure 35b and Supplementary 34).

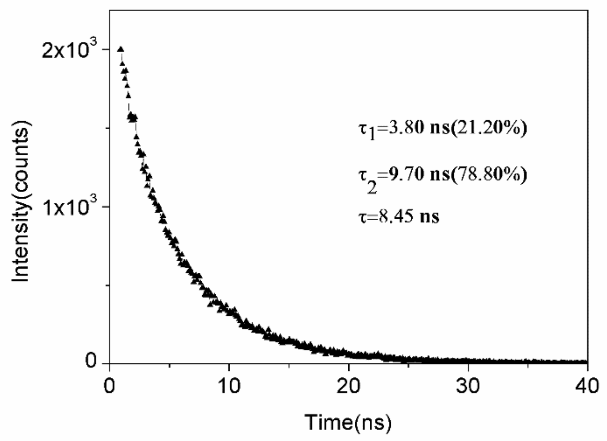

Supplementary Figure 36. PL decay spectra of BT-M in solid state.

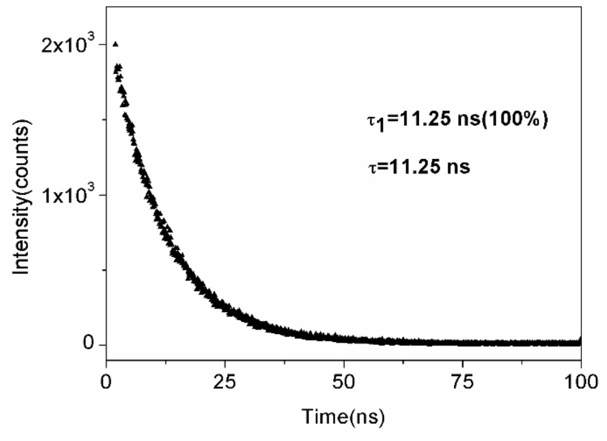

Supplementary Figure 37. PL decay spectra of BT-LC in solid state.

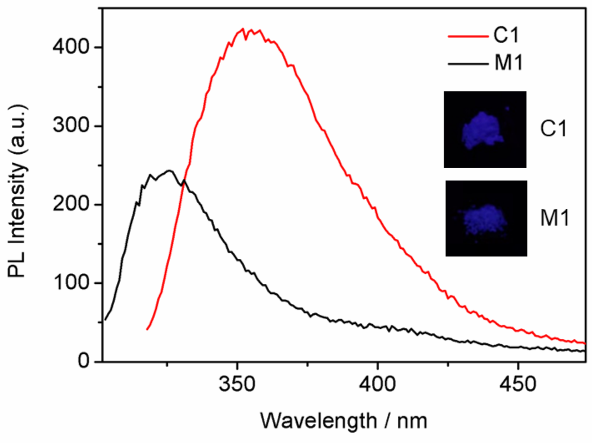

Supplementary Figure 38. PL spectra of C1 and M1 (Insets: photographs in solid state under 365 nm UV illuminations).

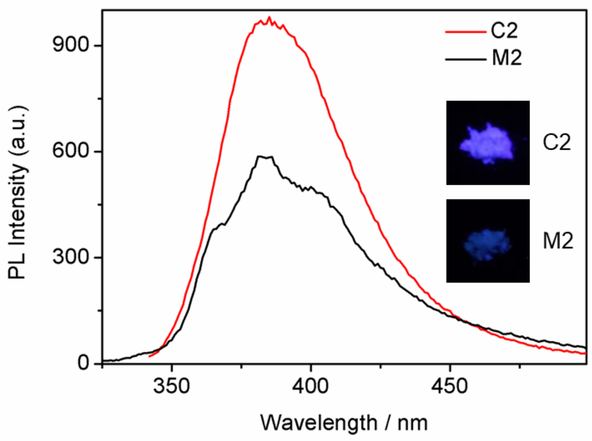

Supplementary Figure 39. PL spectra of C2 and M2 (Insets: photographs in solid state under 365 nm UV illuminations).

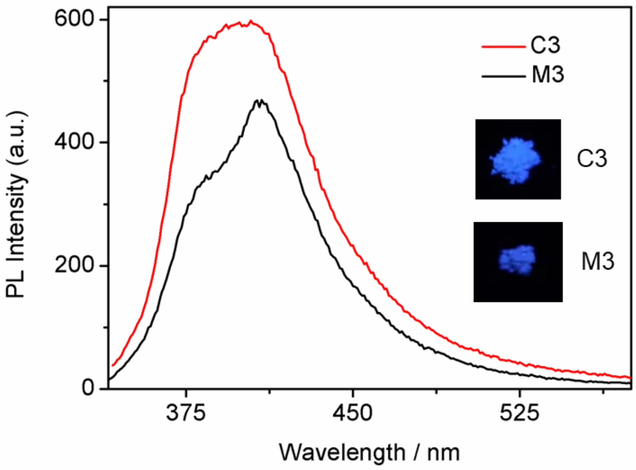

Supplementary Figure 40. PL spectra of C3 and M3 (Insets: photographs in solid state under 365 nm UV illuminations).

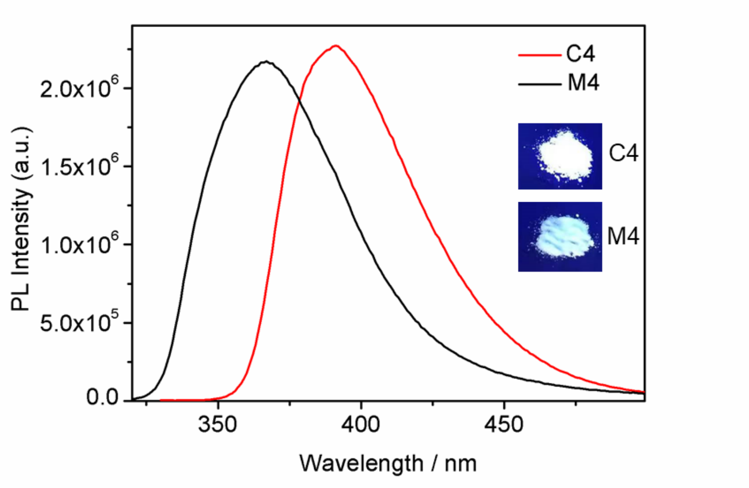

Supplementary Figure 41. PL spectra of C4 and M4 (Insets: photographs in solid state under 365 nm UV illuminations).

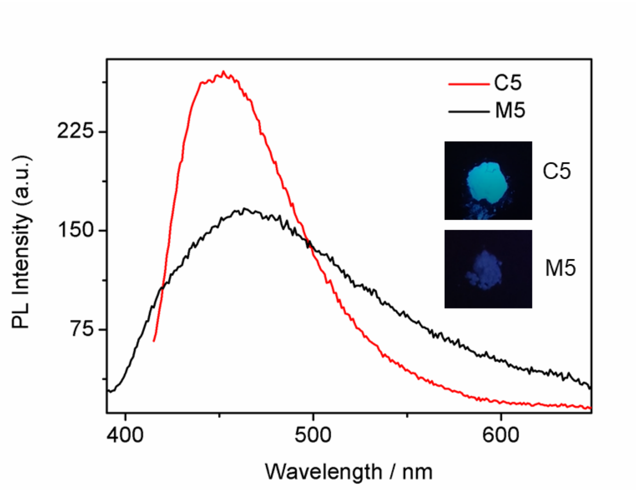

Supplementary Figure 42. PL spectra of C5 and M5 (Insets: photographs in solid state under 365 nm UV illuminations).

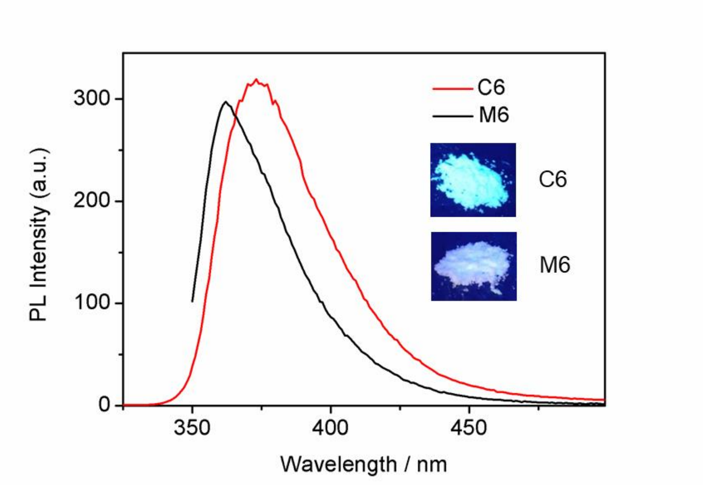

Supplementary Figure 43. PL spectra of C6 and M6 (Insets: photographs in solid state under 365 nm UV illuminations).

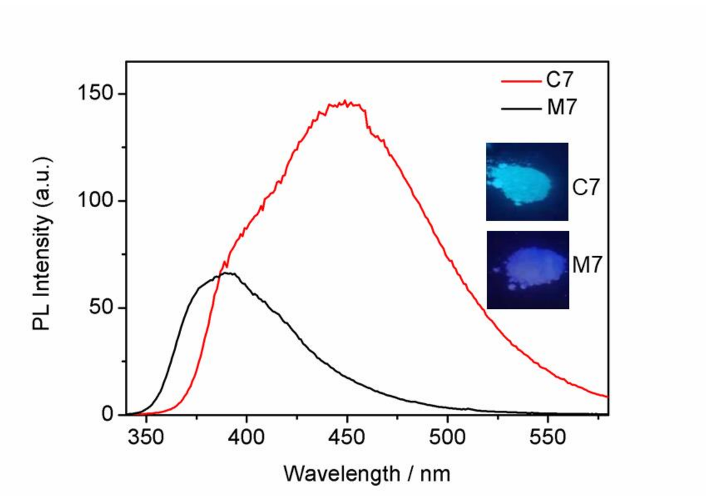

Supplementary Figure 44. PL spectra of C7 and M7 (Insets: photographs in solid state under 365 nm UV illuminations).

2.2 Crystallography data

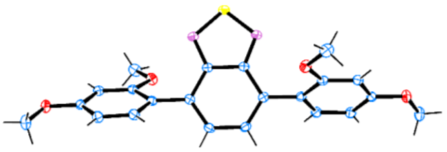

ORTEP drawing of BT-M recorded at 193 K, showing 30% probability thermal ellipsoids.

Supplementary Table 2. Crystal data and structure refinement for BT-M

Identification code	BT-M	
Empirical formula	$C_{22} H_{20} N_2 O_4 S$	
Formula weight	408.46	
Temperature	193(2) K	
Wavelength	0.71073 Å	
Crystal system	Monoclinic	
Space group	C2/c	
Unit cell dimensions	a = 24.495(5) Å	a= 90 °.
	b = 10.9889(19) Å	b= 99.153(6) °.
	c = 7.2661(13) Å	g = 90 °.
Volume	1930.9(6) $Å^3$	
Z	4	
Density (calculated)	1.405 Mg/m^3	
Absorption coefficient	0.200 mm^{-1}	
F(000)	856	
Crystal size	0.180 x 0.120 x 0.100 mm^3	
Theta range for data collection	2.036 to 25.008 °.	
Index ranges	-24<=h<=29, -13<=k<=13, -8<=l<=8	
Reflections collected	5882	
Independent reflections	1643 [R(int) = 0.0451]	
Completeness to theta = 25.008 °	96.5 %	
Refinement method	Full-matrix least-squares on F^2	
Data / restraints / parameters	1643 / 0 / 134	
Goodness-of-fit on F^2	0.977	
Final R indices [I>2sigma(I)]	R1 = 0.0519, wR2 = 0.1605	
R indices (all data)	R1 = 0.0621, wR2 = 0.1712	
Extinction coefficient	n/a	
Largest diff. peak and hole	0.309 and -0.400 e.$Å^{-3}$	
CCDC number	2074796	

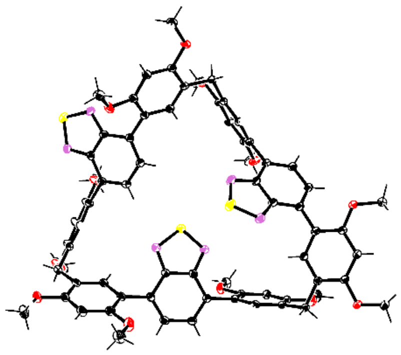

ORTEP drawing of BT-LC recorded at 193 K, showing 30% probability thermal ellipsoids.

Supplementary Table 3. Crystal data and structure refinement for BT-LC

Identification code	BT-LC	
Empirical formula	$C_{71} H_{63} N_7 O_{12} S_3$	
Formula weight	1302.46	
Temperature	193(2) K	
Wavelength	0.71073 Å	
Crystal system	Triclinic	
Space group	P-1	
Unit cell dimensions	a = 12.3202(7) Å	a= 103.891(2)°.
	b = 12.6238(8) Å	b= 91.973(2)°.
	c = 22.5570(14) Å	g = 96.174(2)°.
Volume	3379.3(4) $Å^3$	
Z	2	
Density (calculated)	1.280 Mg/m^3	
Absorption coefficient	0.176 mm^{-1}	
F(000)	1364	
Crystal size	0.180 x 0.170 x 0.110 mm^3	
Theta range for data collection	1.959 to 25.010°.	
Index ranges	-14<=h<=13, -14<=k<=15, -24<=l<=26	
Reflections collected	25128	
Independent reflections	11766 [R(int) = 0.0502]	
Completeness to theta = 25.010°	98.9 %	
Refinement method	Full-matrix least-squares on F^2	
Data / restraints / parameters	11766 / 0 / 851	
Goodness-of-fit on F^2	1.003	
Final R indices [I>2sigma(I)]	R1 = 0.0543, wR2 = 0.1448	
R indices (all data)	R1 = 0.0773, wR2 = 0.1652	
Extinction coefficient	n/a	
Largest diff. peak and hole	0.445 and -0.409 e.$Å^{-3}$	
CCDC number	2074805	

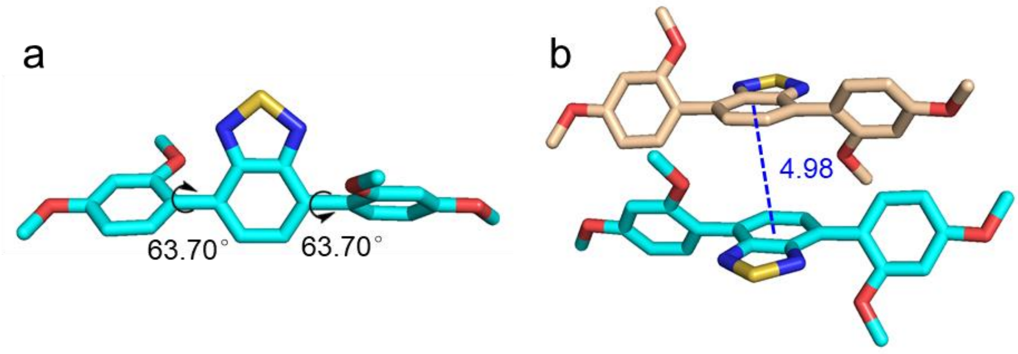

Supplementary Figure 45. Single-crystal X-ray diffraction analysis of BT-M, (a) torsion angles; (b) the stacking arrangement of BT-M. Part of the hydrogen atoms are omitted for clarity.

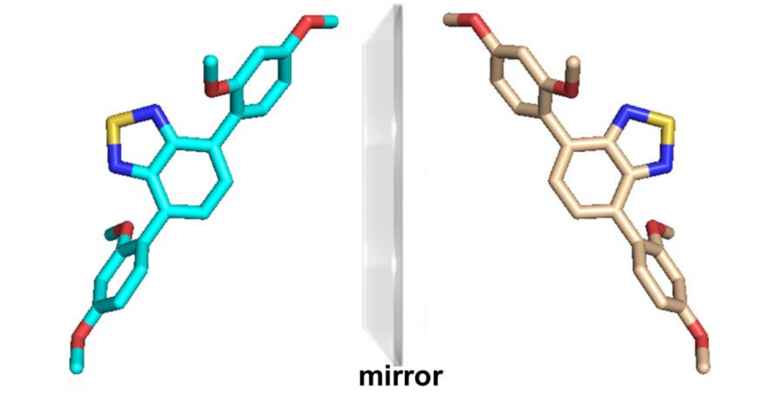

Supplementary Figure 46. A pair of enantiomers in BT-M crystal.

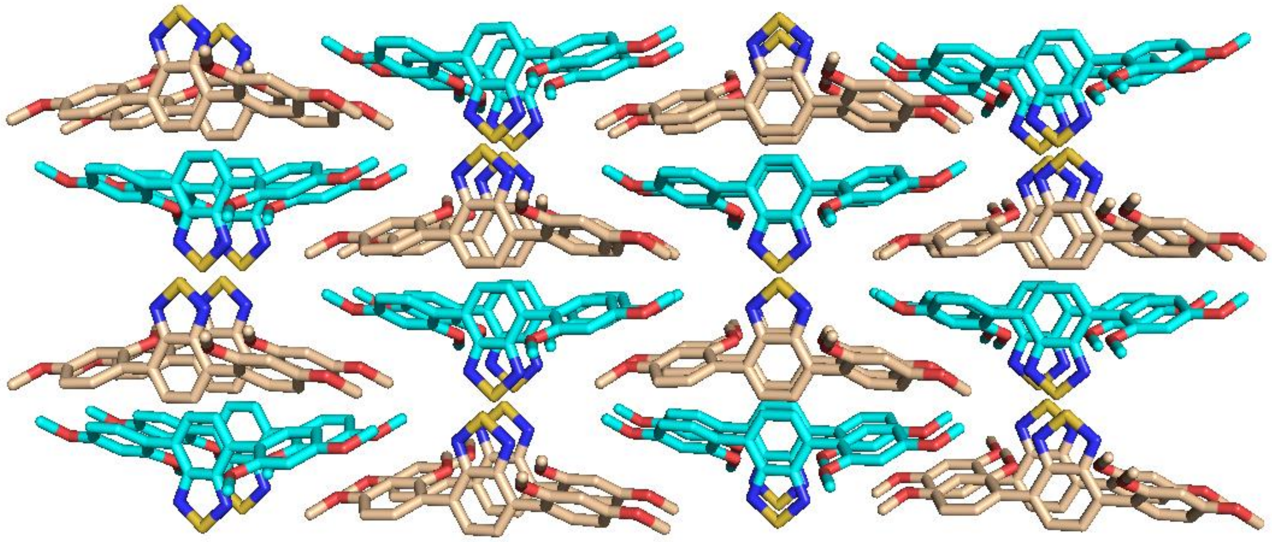

Supplementary Figure 47. The stacking mode of BT-M. The hydrogen atoms are omitted for clarity.

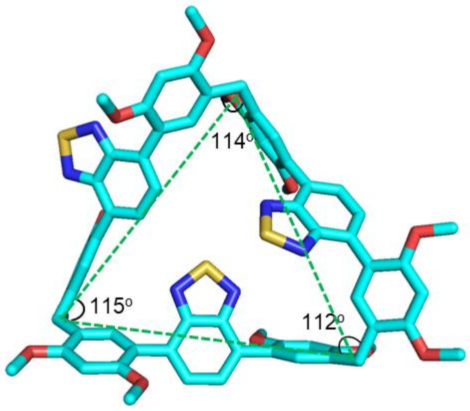

Supplementary Figure 48. Single-crystal X-ray diffraction analysis of BT-LC. Part of the hydrogen atoms are omitted for clarity.

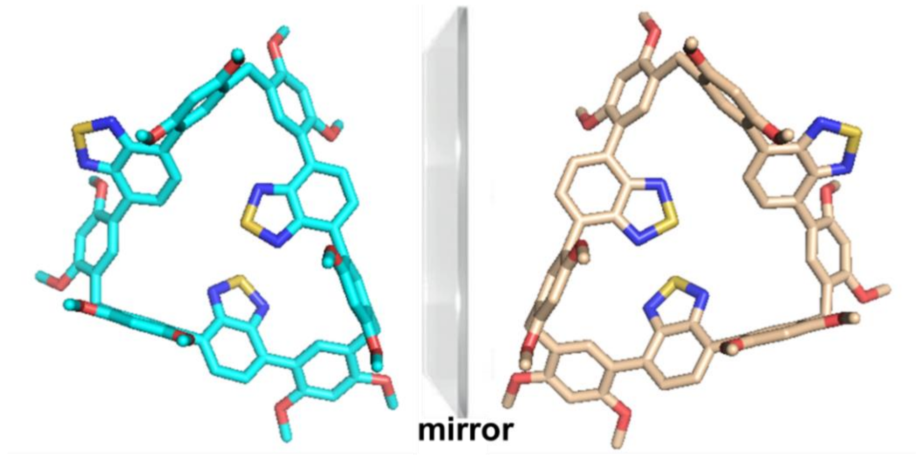

Supplementary Figure 49. A pair of enantiomers in BT-LC crystal. The hydrogen atoms are omitted for clarity.

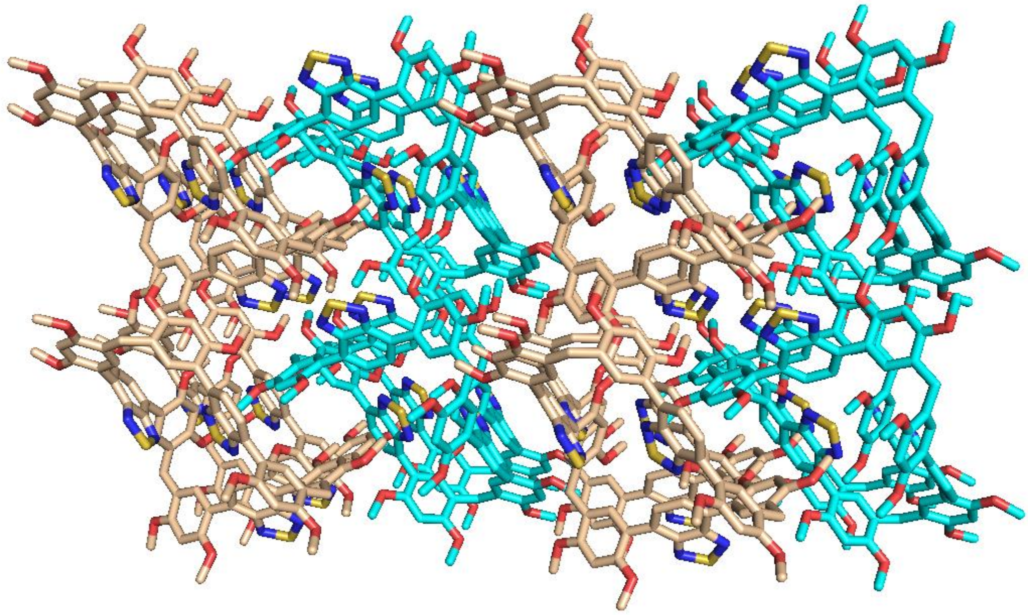

Supplementary Figure 50. The stacking mode of BT-LC. The hydrogen atoms and sovents are omitted for clarity.

2.3 Theoretical calculation

Quantum chemical calculation methods

All the calculations of ground states were performed at the PBE0/6-31g* level[11] using the Gaussian16 suite of programs.[12] For excited state calculation, The Tamm-Dancoff approximation (TDA)[13] is used for TDDFT because it is more stable near minimum energy crossing point (MECP).[14] Harmonic vibration frequency calculations are used to confirm the stationary points. $MECP_{S1/S0}$ is located at the TDA-PBE0/PBE0/6-31G* level using the Newton-Lagrange method, which was introduced by Koga and Morokuma.[15] These calculations are treated using a homemade program LookForMECP (version 2.1). This program can be obtained from the authors upon request. The early version of this program has been used successfully to search the MECP.[16] The 3D figures of molecular structure were prepared by CYLView.[17]

Coordinates (Å) and energies (Hartree)

$S0_{min}$

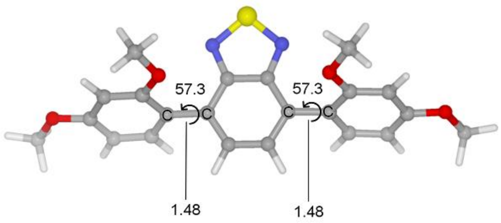

E = -1657.336726 hartree

C	0.62416600	-1.29102100	0.13084600
C	-0.71323000	-1.28214100	0.20256300
C	1.39082400	-0.06492000	0.36866700
C	-1.40919900	-0.00794700	0.21488500
N	-1.36483300	-2.59721700	0.10475600
N	1.22798300	-2.62410200	-0.04674200
C	0.67565300	1.16800000	0.62509200
C	2.79846900	-0.03149100	0.25108400
C	-0.66501200	1.19953200	0.51767500
C	-2.80061900	0.08126200	-0.04765500

S	-0.02073200	-3.31610100	0.80461600
H	1.21697800	2.07610500	0.83790400
C	3.44881900	-1.11635000	-0.43584200
C	3.67073400	1.06531900	0.66854100
H	-1.19538500	2.13039600	0.65709400
C	-3.61927300	-1.07960900	0.15523900
C	-3.49446800	1.29135300	-0.46523400
C	4.73678400	-1.03446200	-0.88981900
H	2.83104100	-2.01221500	-0.56714900
C	4.96344900	1.14495100	0.19862700
O	3.18121500	1.94252800	1.54982700
C	-4.98892000	-1.03095000	0.11140800
H	-3.05786900	-1.99865000	0.36724700
C	-4.87196200	1.33375500	-0.49557300
O	-2.74028400	2.32021700	-0.87209600
C	5.49402500	0.11940100	-0.60665000
H	5.16577200	-1.86579200	-1.43658400
H	5.62398600	1.96114500	0.46141400
C	4.01265100	2.98933300	2.01473200
C	-5.62573700	0.18547500	-0.19058200
H	-5.56150900	-1.92894400	0.31156100
H	-5.41660200	2.22126300	-0.79129300
C	-3.37640900	3.49641100	-1.33215400
O	6.74835300	0.31466000	-1.00323800
H	4.90246100	2.58835500	2.51331900
H	4.31419200	3.65234800	1.19534300
H	3.40941100	3.54473800	2.73286700
O	-6.94662600	0.35481700	-0.24799300
H	-4.00478800	3.28499500	-2.20520500
H	-3.98343500	3.95750500	-0.54399500

H	-2.57065600	4.17321500	-1.61725400
C	7.38520100	-0.67865700	-1.79692700
C	-7.79300100	-0.75846800	0.00129400
H	7.47975400	-1.61850000	-1.24405200
H	6.83547700	-0.84653100	-2.72829700
H	8.37558700	-0.28239900	-2.01982400
H	-7.62178300	-1.55315200	-0.73194000
H	-7.64153000	-1.14732700	1.01350200
H	-8.81037200	-0.38012600	-0.09791500

$S1_{min}$

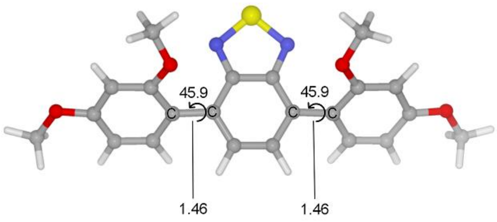

E = -1657.235636 hartree

C	1.42952000	-0.60458200	0.23031300
C	0.69347800	-1.66353500	0.79348900
C	0.71473200	0.45474800	-0.41033000
C	-0.69331400	-1.66364200	0.79352600
H	1.22788500	-2.46228500	1.30045400
C	2.88463000	-0.66117100	0.23172800
C	-0.71492200	0.45464700	-0.41027800
N	1.28355800	1.44487000	-1.12789000
C	-1.42952400	-0.60477500	0.23041400
H	-1.22760000	-2.46248000	1.30048600
C	3.55286200	-1.85321500	-0.08548900
C	3.69649400	0.47944500	0.52599600

N	-1.28397100	1.44467700	-1.12777500
S	-0.00029400	2.32234000	-1.74064700
C	-2.88461200	-0.66143300	0.23187600
C	4.92961700	-1.92752000	-0.21119200
H	2.95281700	-2.73112500	-0.30627600
C	5.07615100	0.42854200	0.37256300
O	3.04811300	1.53798000	1.01199800
C	-3.55296100	-1.85343100	-0.08521600
C	-3.69638000	0.47933000	0.52604500
C	5.69477000	-0.76670500	0.00008100
H	5.39562500	-2.86283500	-0.49843200
H	5.70597300	1.28721100	0.57182100
C	3.70339500	2.78642800	1.03737000
C	-4.92971600	-1.92753800	-0.21099500
H	-2.95301600	-2.73144500	-0.30586400
C	-5.07602900	0.42863300	0.37244300
O	-3.04788300	1.53770100	1.01217800
O	7.03623100	-0.71415500	-0.11061500
H	4.51966700	2.79647200	1.77148100
H	4.09021900	3.04025800	0.04438400
H	2.94406100	3.51226500	1.32865300
C	-5.69475100	-0.76658100	0.00005200
H	-5.39584300	-2.86281600	-0.49816700
H	-5.70572100	1.28742700	0.57156700
C	-3.70277800	2.78634500	1.03727200
C	7.72968800	-1.89046900	-0.47065800
O	-7.03617600	-0.71391700	-0.11083100
H	-4.08935000	3.04017300	0.04418800
H	-4.51917000	2.79672300	1.77124900
H	-2.94327900	3.51200000	1.32857800

H	7.58291300	-2.68518200	0.27111900
H	7.41955800	-2.25105400	-1.45899300
H	8.78492200	-1.61603700	-0.50146000
C	-7.72977700	-1.89029400	-0.47040900
H	-7.41986000	-2.25117100	-1.45870400
H	-7.58290900	-2.68479800	0.27156800
H	-8.78499600	-1.61579300	-0.50108700

$MECP_{S1/S0}$

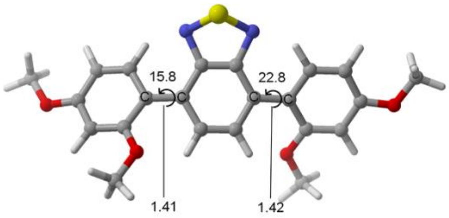

E = -1657.157554 hartree

C	0.62416600	-1.29102100	0.13084600
C	-0.71323000	-1.28214100	0.20256300
C	1.39082400	-0.06492000	0.36866700
C	-1.40919900	-0.00794700	0.21488500
N	-1.36483300	-2.59721700	0.10475600
N	1.22798300	-2.62410200	-0.04674200
C	0.67565300	1.16800000	0.62509200
C	2.79846900	-0.03149100	0.25108400
C	-0.66501200	1.19953200	0.51767500
C	-2.80061900	0.08126200	-0.04765500
S	-0.02073200	-3.31610100	0.80461600
H	1.21697800	2.07610500	0.83790400
C	3.44881900	-1.11635000	-0.43584200
C	3.67073400	1.06531900	0.66854100

H	-1.19538500	2.13039600	0.65709400
C	-3.61927300	-1.07960900	0.15523900
C	-3.49446800	1.29135300	-0.46523400
C	4.73678400	-1.03446200	-0.88981900
H	2.83104100	-2.01221500	-0.56714900
C	4.96344900	1.14495100	0.19862700
O	3.18121500	1.94252800	1.54982700
C	-4.98892000	-1.03095000	0.11140800
H	-3.05786900	-1.99865000	0.36724700
C	-4.87196200	1.33375500	-0.49557300
O	-2.74028400	2.32021700	-0.87209600
C	5.49402500	0.11940100	-0.60665000
H	5.16577200	-1.86579200	-1.43658400
H	5.62398600	1.96114500	0.46141400
C	4.01265100	2.98933300	2.01473200
C	-5.62573700	0.18547500	-0.19058200
H	-5.56150900	-1.92894400	0.31156100
H	-5.41660200	2.22126300	-0.79129300
C	-3.37640900	3.49641100	-1.33215400
O	6.74835300	0.31466000	-1.00323800
H	4.90246100	2.58835500	2.51331900
H	4.31419200	3.65234800	1.19534300
H	3.40941100	3.54473800	2.73286700
O	-6.94662600	0.35481700	-0.24799300
H	-4.00478800	3.28499500	-2.20520500
H	-3.98343500	3.95750500	-0.54399500
H	-2.57065600	4.17321500	-1.61725400
C	7.38520100	-0.67865700	-1.79692700
C	-7.79300100	-0.75846800	0.00129400
H	7.47975400	-1.61850000	-1.24405200

H	6.83547700	-0.84653100	-2.72829700
H	8.37558700	-0.28239900	-2.01982400
H	-7.62178300	-1.55315200	-0.73194000
H	-7.64153000	-1.14732700	1.01350200
H	-8.81037200	-0.38012600	-0.09791500

2.4 TGA of BT-M and BT-LC

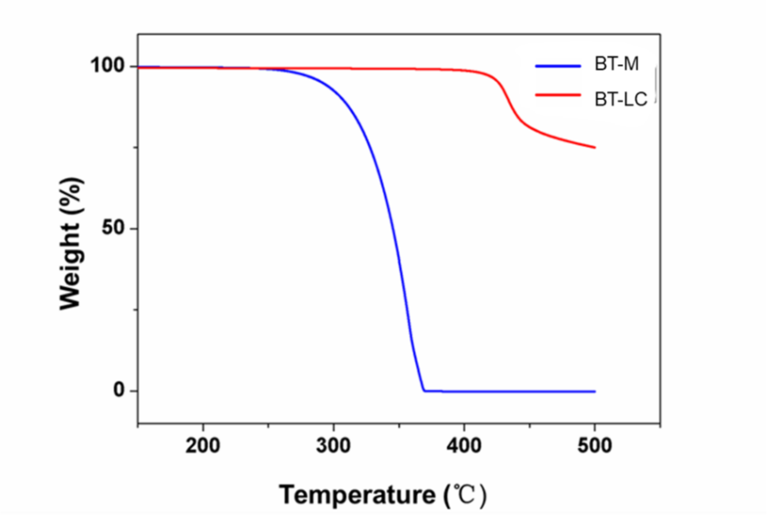

Supplementary Figure 51. TGA profile of BT-M and BT-LC recorded under a nitrogen atmosphere.

2.5 Electroluminescence

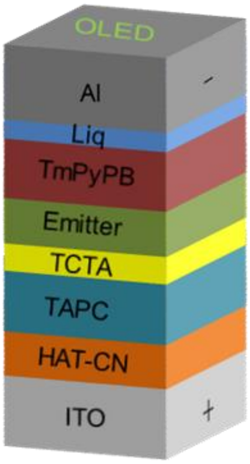

Supplementary Figure 52. Schematic diagram of the devices.

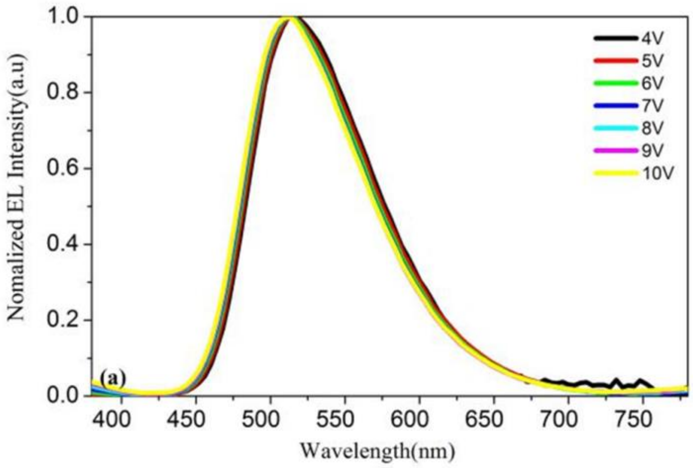

Supplementary Figure 53. EL spectra of device A.

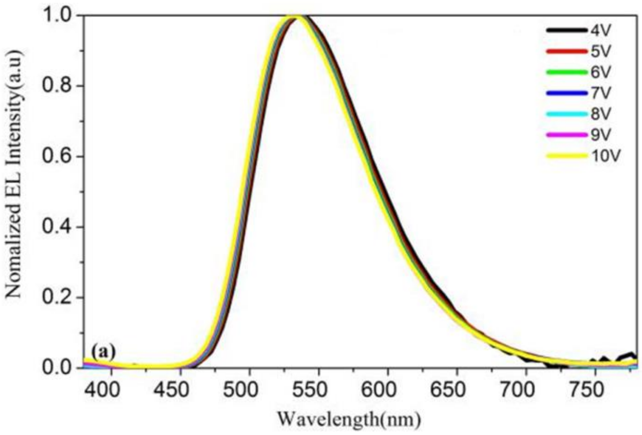

Supplementary Figure 54. EL spectra of device B.

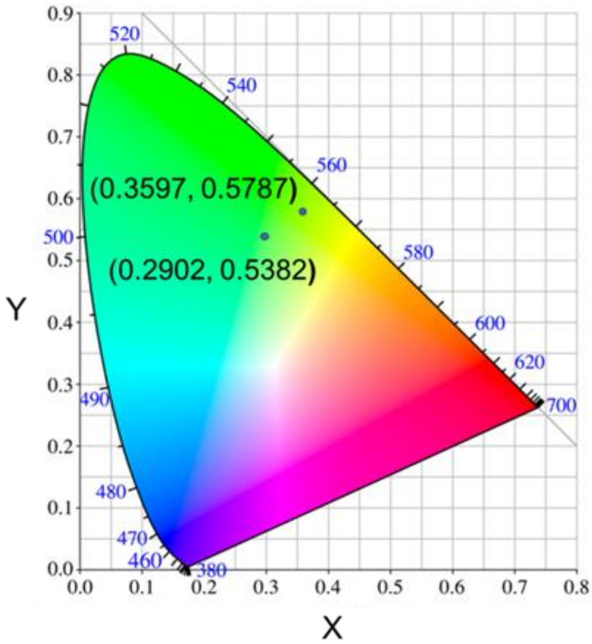

Supplementary Figure 55. CIE chromaticity coordinates of device A and B.

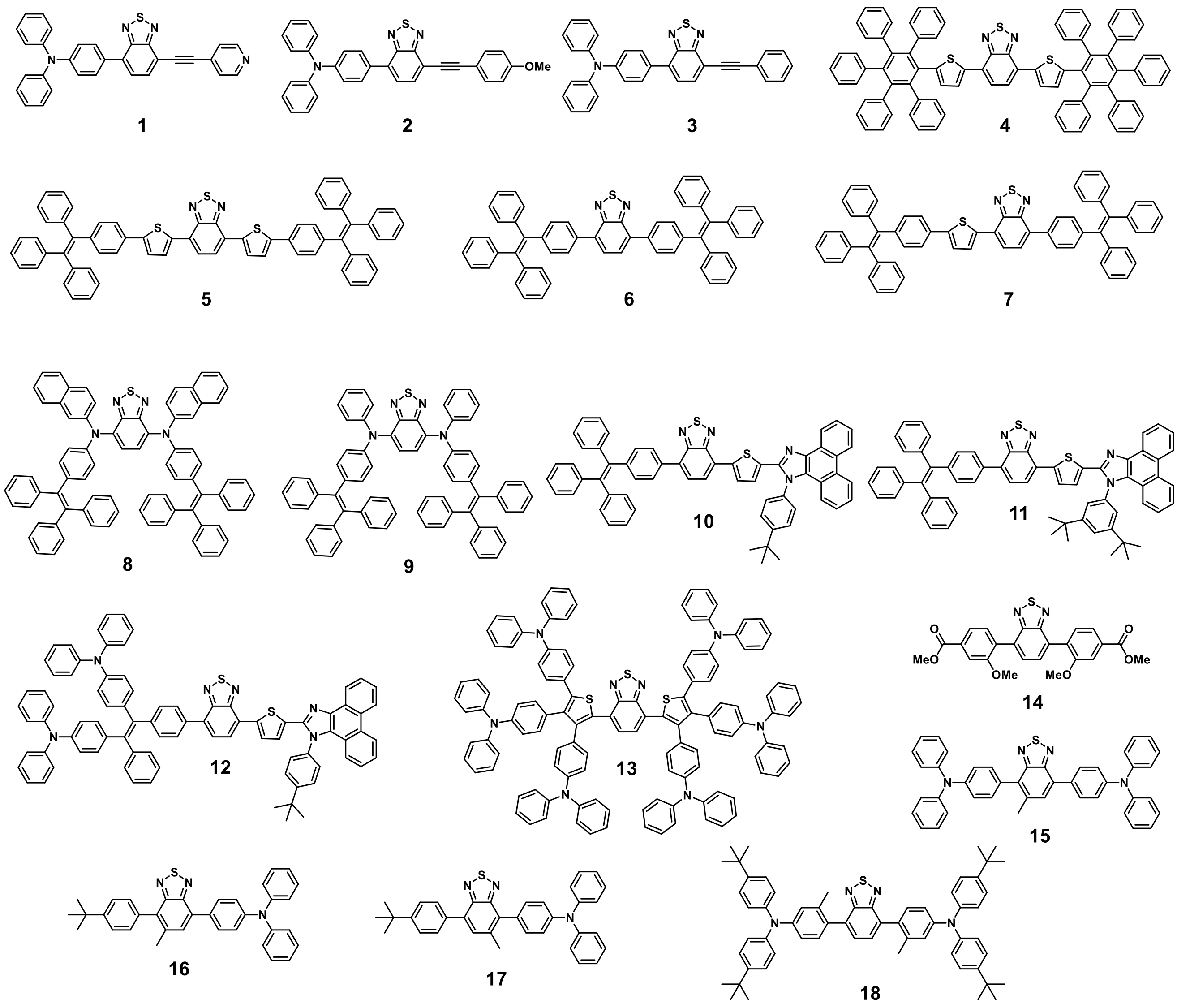

Supplementary Figure 56. Chemical structures of benzothiadiazole-based emitters.

Supplementary Table 4. Electroluminescence properties of benzothiadiazole-based emitters

	CE_{max} (cd A^{-1})	PE_{max} (lm W^{-1})	EQE_{max} (%)	Ref.
BT-M	10.1	7.10	1.92	This work
BT-LC	9.93	8.25	2.82	This work
1	0.28	0.15	0.15	18
2	0.68	0.51	0.32	18
3	0.88	0.64	0.40	18
4	1.37	-[a]	1.00	19
5	0.4	0.5	1.00	20
6	5.2	3.0	1.50	20
7	6.4	2.9	3.10	20

8	-[a]	-[a]	1.43	21
9	-[a]	-[a]	1.73	21
10	1.31	1.59	2.17	22
11	1.41	1.70	2.03	22
12	2.19	1.61	2.09	22
13	6.25	5.17	-[a]	23
14	6.5	2.6	2.39	24
15	6.2	11.6	4.5	25
16	15.7	12.2	4.6	25
17	15.2	10.9	4.8	25
18	30.4	23.67	8.47	26

"a" means that this value is not given in the original reference.

3. Supplementary References

1. Wang, Y., Xu, K., Li, B., Cui, L., Li, J., Jia, X., Zhao, H., Fang, J., Li, C., Efficient Separation of cis- and trans-1,2-Dichloroethene Isomers by Adaptive Biphen[3]arene Crystals. Angew. Chem. Int. Ed. **58**, 10281-10284 (2019).
2. Xu, K., Zhang, Z., Yu, C., Wang, B., Dong, M., Zeng, X., Gou, R., Cui, L., Li, C. J. A Modular Synthetic Strategy for Functional Macrocycles. Angew. Chem. Int. Ed. **59**, 7214-7218 (2020).
3. Mei, J.; Leung, N. L. C.; Kwok, R. T. K.; Lam, J. W. Y.; Tang, B. Z. Aggregation-Induced Emission: Together We Shine, United We Soar! *Chem. Rev.* **115,** 11718-11940 (2015).
4. Morozumi, T., Anada, T. Nakamura, H. New Fluorescent "Off-On" Behavior of 9-Anthryl Aromatic Amides through Controlling the Twisted Intramolecular Charge Transfer Relaxation Process by Complexation with Metal Ions. J. Phys. Chem. B **105**,2923-2931 (2001).
5. Sasaki, S., Drummen, G. P. C., Konishi, G. i. Recent advances in twisted intramolecular charge transfer (TICT) fluorescence and related phenomena in materials chemistry. J. Mater. Chem. C **4**, 2731-2743 (2016).
6. Karmakar, S., Ambastha, A., Jha, A., Dharmadhikari, A., Dharmadhikari, J., Venkatramani, R., Dasgupta, J. Transient Raman Snapshots of the Twisted Intramolecular Charge Transfer State in a Stilbazolium Dye. J. Phys. Chem. Lett. **11**, 4842-4848 (2020).
7. Yan, X., Cook, T. R., Wang, P., Huang, F., Stang, P. J. Highly emissive platinum (II) metallacages. Nat. Chem. **7 (4)**, 342-348 (2015).
8. Zheng, X., Zhu, W., Zhang, C., Zhang, Y., Zhong, C., Li, H., Xie, G., Wang, X., Yang, C. Self-Assembly of a Highly Emissive Pure Organic Imine-Based Stack for Electroluminescence and Cell

Imaging. J. Am. Chem. Soc. **141**, 4704-4710 (2019).

9. Chen, G., Li, W. B., Zhou, T. R., Peng, Q., Zhai, D., Li, H. X., Yuan, W. Z., Zhang, Y. M., Tang, B. Z. Conjugation-Induced Rigidi-ty in Twisting Molecules: Filling the Gap Between Aggregation-Caused Quenching and Aggregation-Induced Emission. Adv. Mater. **27**, 4496-4501 (2015).
10. Xu, Y., Ren, L., Dang, D., Zhi, Y., Wang, X., Meng, L., A Strategy of “Self-Isolated Enhanced Emission” to Achieve Highly Emissive Dual-State Emission for Organic Luminescent Materials, Chem. Eur. J. **24**, 10383-10389 (2018).
11. (a) Perdew, J. P., Burke, K., Ernzerhof. M. Generalized gradient approximation made simple. Phys. Rev. Lett. **77**, 3865-3868 (1996); (b) Perdew, J. P., Burke, K., Ernzerhof. M. Errata: Generalized gradient approximation made simple. Phys. Rev. Lett. **78**, 1396-1396 (1997); (c) Adamo, C., Barone, V. Toward reliable density functional methods without adjustable parameters: The PBE0 model. J. Chem. Phys. **110**, 6158-6169 (1999).
12. Frisch, M. J. *et al.* Gaussian 16 revision A.03 (Gaussian Inc., 2019).
13. Dreuw, A., Head-Gordon, M. Single-Reference ab Initio Methods for the Calculation of Excited States of Large Molecules. Chem. Rev. **105**, 4009-4037 (2005).
14. Matsika, S. Electronic Structure Methods for the Description of Nonadiabatic Effects and Conical Intersections. Chem. Rev. **121**, 9407-9449 (2021).
15. Koga, N., Morokuma, K. Determination of the lowest energy point on the crossing seam between two potential surfaces using the energy gradient. Chem. Phys. Lett. **119**, 371-374 (1985).
16. (a) Zhao, H., Bian, W., Liu, K. A theoretical study of the reaction of $O(^3P)$ with isobutene. J. Phys. Chem. A **110**, 7858-7866 (2006); (b) Zhao, S., Wu, W., Zhao, H., Wang, H., Yang, C., Liu, K., Su, H. Adiabatic and nonadiabatic reaction pathways of the $O(^3P)$ with propyne. J. Phys. Chem. A **113**, 23-34 (2009); (c) Liu, K., Li, Y., Su, J., Wang, B. The reliability of DFT methods to predict electronic structures and minimum energy crossing point for $[Fe^{IV}O](OH)_2$ models: A comparison study with MCQDPT method. J. Comput. Chem. **35**, 703-710 (2014); (d) Li, H., Li, D., Zeng, X., Liu, K., Beckers, H., Schaefer, H. F. III., Esselman, B. J., McMahon, R. J. Toward Understanding the Decomposition of Carbonyl Diazide $(N_3)_2C{=}O$ and Formation of Diazirinone cycl-N2CO: Experiment and Computations J. Phys. Chem. A **119**, 8903-8911 (2015); (e) Wu, Z., Feng, R., Li, H., Xu, J., Deng, G., Abe, M., Begue, D., Liu, K., Zeng, X Fast Heavy-Atom Tunneling in Trifluoroacetyl Nitrene. Angew. Chem. Int. Ed. **65**, 15672-15676 (2017).
17. CYLview20; Legault, C. Y., Université de Sherbrooke, 2020 (http://www.cylview.org)
18. Peng, Z., Zhang, K., Huang, Z., Wang, Z., Duttwyler, S., Wang, Y., Lu, P., Emissions from a triphenylamine–benzothiadiazole–monocarbaborane triad and its applications as a fluorescent chemosensor and a white OLED component. J Mater Chem C, **7**, 2430-2435 (2019).
19. Sun, X., Xu, X., Qiu, W., Yu, G., Zhang, H., Gao, X., Chen, S., Song, Y., Liu, Y., A non-planar

pentaphenylbenzene functionalized benzo[2,1,3]thiadiazole derivative as a novel red molecular emitter for non-doped organic light-emitting diodes. J Mater Chem, **18**, 2709 (2008).

20. Zhao, Z., Deng, C., Chen, S., Lam, JWY., Qin, W., Lu, P., Wang, Z., Kwok, HS., Ma, Y., Qiu, H., Tang, BZ., Full emission color tuning in luminogens constructed from tetraphenylethene, benzo-2,1,3-thiadiazole and thiophene building blocks. Chem Commun, **47**, 8847-8849 (2011).
21. Lee, WWH., Zhao, Z., Cai, Y., Xu, Z., Yu, Y., Xiong, Y., Kwok, RTK., Chen, Y., Leung, NLC., Ma, D., Lam, JWY., Qin, A., Tang, BZ., Facile access to deep red/near-infrared emissive AIEgens for efficient non-doped OLEDs. Chem Sci, **9**, 6118-6125 (2018).
22. Li, Y., Wang, W., Zhuang, Z., Wang, Z., Lin, G., Shen, P., Chen, S., Zhao, Z., Tang, BZ., Efficient red AIEgens based on tetraphenylethene: synthesis, structure, photoluminescence and electroluminescence. J Mater Chem C, **6**, 5900-5907 (2018).
23. Thangthong, A., Prachumrak, N., Sudyoadsuk, T., Namuangruk, S., Keawin, T., Jungsuttiwong, S., Kungwan, N., Promarak, V., Multi-triphenylamine–functionalized dithienylbenzothiadiazoles as hole-transporting non-doped red emitters for efficient simple solution processed pure red organic light-emitting diodes. Org Electron, **21**, 117-125 (2015).
24. Angioni, E., Chapran, M., Ivaniuk, K., Kostiv, N., Cherpak, V., Stakhira, P., Lazauskas, A., Tamulevius, S., Volyniuk, D., Findlay, NJ., Tuttle, T., Grazulevicius, JV., Skabara, PJ., A single emitting layer white OLED based on exciplex interface emission. J Mater Chem C, **4**, 3851-3856 (2016).
25. Pathak, A., Justin Thomas, KR., Singh, M., Jou, JH., Fine-Tuning of Photophysical and Electroluminescence Properties of Benzothiadiazole-Based Emitters by Methyl Substitution. J Org Chem, **82**, 11512-11523 (2017).
26. Zhang, Y., Zhou, X., Zhou, C., Su, Q., Chen, S., Song, J., Wong, W.-Y., High-efficiency organic electroluminescent materials based on the D-A-D type with sterically hindered methyl groups. J. Mater. Chem. C, **8**, 6851-6860 (2020).
